# Interpreting in healthcare: a systematic search and mapping of ethical issues in health literature

**DOI:** 10.3389/fpubh.2026.1856115

**Published:** 2026-08-18

**Authors:** Sonakshi Bhalla, Ana S. Iltis, Jordan Traylor, Kirstin R. W. Matthews

**Affiliations:** 1 Baker Institute for Public Policy, Science and Technology Policy Program, Rice University, Houston, TX, United States; 2 Department of Philosophy and Center for Bioethics, Health and Society, Wake Forest University, Winston-Salem, NC, United States

**Keywords:** equity, language proficiency, medical interpretation, moral distress, translation

## Abstract

Medical interpreters play a vital role in healthcare settings, ensuring effective communication between providers and their patients when language barriers exist. Interpreters can be *ad hoc*, such as family members, or individuals who have undergone formal training. We systematically searched and analyzed 112 medical, bioethics, and interpreting literature publications to answer the question: what ethical issues arise when trained medical interpreters are involved in patient care? Interpreting in a medical setting is a complex process that requires not just understanding medical terminology in two languages, but also potentially helping to bridge cultures and serving as a patient advocate. These roles sometimes conflict, which can lead to numerous ethics issues and questions. We grouped these ethical concerns into categories, including accuracy, neutrality, confidentiality, professionalism, interprofessionalism (working with the healthcare team), and time constraints. Our findings expose the complex ethical challenges that medical interpreters face in conducting their work, which give rise to moral and emotional distress. These ethical concerns require additional research and attention not only in educating interpreters but in training other health professionals and administrators and developing health systems policies.

## Introduction

Language barriers in healthcare settings can impede effective communication between providers and their patients. Language and communication discordance between clinicians and patients is common in the United States. Roughly 27.5 million U.S. residents have limited English proficiency and 37.5 million have difficulty hearing (1). Other high-income countries with significant immigrant populations likely will also face many cases of communication gaps in healthcare. For instance, European data indicate that over 10% of people born outside of the country in which they are living have only an intermediate grasp of their “host country's” language and slightly over 3% have no grasp of the language (2).

When a mismatch occurs between a patient and clinician, an interpreter may be necessary to facilitate communication. Interpretation refers to “a purposeful approach to communication that facilitates meaningful, relevant, and inclusive experiences that deepen understanding, broaden perspectives, and inspire engagement with the world around us” (3). In the healthcare context, interpreters “facilitate accurate exchange of information, ensuring that both the physician's and the patient's voices are clearly heard and understood” (4).

Broadly speaking, there are two categories of individuals who interpret in healthcare settings: trained interpreters and *ad hoc* interpreters (5–9). The first group are individuals who have gone through a program to understand the role of medical interpreters, the ethical standards for interpreting, and relevant terminology, and to develop interpreting skills and techniques. Such programs prepare individuals to serve as “qualified interpreters.” The Affordable Care Act defines a “qualified interpreter” as someone who adheres to accepted interpreter ethics principles, has demonstrated proficiency in speaking and understanding both English and the target language, and is able to interpret effectively, accurately, and impartially using any necessary vocabulary (45 C.F.R. § 92.4). Some trained medical interpreters have completed a rigorous curriculum and degree. Others are individuals, such as healthcare personnel, students, or community members, who undergo basic medical interpreter training, such as through a certificate program. Trained interpreters may volunteer their services or be paid.

*Ad hoc* or informal interpreters are individuals who are able to communicate in the language of a patient and clinician but are not formally trained in medical interpreting. Individuals who may serve as *ad hoc* interpreters include family members, friends, bystanders, or other healthcare providers or staff in a healthcare facility (5–9). Concerns associated with the use of *ad hoc* interpreters include increased risk of error and threats to patient safety, greater likelihood that patients will withhold information such as physical or mental health symptoms, lower likelihood of the patient being referred for specialty care, violations of patient privacy and confidentiality, higher risk of hospital readmission, lower rate of adherence to recommended treatment, and lower patient satisfaction (5–13). It is widely, though not universally, held that *ad hoc* interpreters should not typically be used in healthcare, because of the previously cited concerns (5–9). However, there are situations in which patients are reluctant to use trained interpreters, preferring to rely on friends, and respect for the patient's authority over themselves and their care may require respecting this decision (14).

Given the importance of effective communication in healthcare settings, the complex role interpreters play, and the number of patient-clinician pairs who may experience communication mismatches, it is reasonable to expect ethical issues to arise when interpreters are involved in patient care. Many articles in the bioethics, medical, and interpreting literature address concerns associated with communication barriers in healthcare settings, including disparities in care and outcomes that may arise when clinicians and patients do not share a language and the importance of providing trained interpreter services to address communication mismatches (10, 15–19).

The literature has paid less attention to ethical issues that arise in interpreter-assisted care when these interactions involve trained interpreters (4, 20–26). Previous reviews, summarized in Table S1 in Appendix S1, have focused on quality of care for patients who are not proficient in the dominant local language (11–13, 27–32), relational issues, including interprofessional relations (33, 34), and models of healthcare interpretation (35). Some briefly address ethical concerns explicitly (33). Others mention ethically relevant concerns but do not label them as such (28). To our knowledge, there has been no systematic examination of the medical, bioethics, and interpreting literature to identify the ethical issues and questions that arise when trained interpreters are involved in healthcare interactions. We utilized the methods of a systematic search and a mapping review to fill this gap (36). We focused on encounters that involve trained medical interpreters and excluded discussions related to the use of *ad hoc* interpreters because the latter differ in important ways and merit separate consideration, as noted above.

Our results highlight three types of roles that interpreters play (linguistic conduit, cultural broker, and patient advocate) and six discrete categories of ethical issues that arise in the context of medical interpretation: accuracy, neutrality, confidentiality, professionalism, interprofessionalism, and time constraints. In discussing our findings regarding interpreter roles and the categories of ethical concern, an additional theme that cut across many of these emerged, namely interpreter emotional and moral distress. We added this as a seventh ethical issue category and explore this theme separately in the discussion.

## Methods

### Search criteria

Because of the nature of our research question and the literature we wanted to include, we did not conduct a traditional systematic review. However, we adhered to systematic review practices to improve the reliability of our literature search and the quality of our findings. We followed PRISMA reporting guidelines (see Appendix S2 for PRISMA Checklist) (37). The protocol was registered on Open Science Framework on March 24, 2024 (osf.io/xg6yr). In consultation with a librarian from the Z. Smith Reynolds Library at Wake Forest University, Colleen Foy, MLIS, we designed a search strategy that included two databases: PubMed (https://pubmed.ncbi.nlm.nih.gov/) and MLA International Bibliography (https://www.mla.org). We selected these databases because they cover a wide range of publications in medicine, health systems, bioethics, language, and communication. We conducted multiple test searches using additional databases, including Web of Science, CINAHL, and Embase, and did not find additional relevant sources. Thus, we limited our search to these two databases. Appendix S3 includes the search strategy for each database. No date limits were used. Language limits were applied to include only publications in English in PubMed. Searches were completed on April 12, 2024.

We imported search results into Zotero for de-duplication and then to Covidence for screening, selection, and data extraction. We reviewed the reference sections of included publications for additional possible publications to consider. At the time of the search, an issue of *Narrative Inquiry in Bioethics* that included a collection of stories from people who had provided interpreting services in healthcare settings was forthcoming and was included for screening as well. Consistent with published recommendations, we included abstracts (38) and dissertations (39). We also included commentaries, editorials, and other types of publications, such as reports, to maximize data collection.

The gray literature search strategy (outlined in Appendix S4) was developed in consultation with Colleen Foy, MLIS from Z. Smith Reynolds Library. Websites of interpreter societies, government entities with healthcare oversight, and healthcare blogs were identified in consultation with trained interpreters, a reference librarian, and independent research. We added gray literature items that met the initial criteria associated with Title and Abstract Screening to Zotero and entered them into Covidence. We included a total of 16 websites, listed in Appendix S5. Final screening and data extraction were completed in Covidence.

To be eligible, publications had to:

1. Be discoverable using our search strategy.
2. Be published in English.
3. Be available in full text to us online or in print through the Wake Forest University Library, Rice University Library, or through Interlibrary Loan.
4. Identify at least one ethical issue related to interpreting in a healthcare context when the person doing the interpreting is trained to do this work.

Publications were excluded based on failure to meet the highest ranking exclusion criterion, which corresponded to the eligibility criteria above.

### Selection

**Figure 1:**
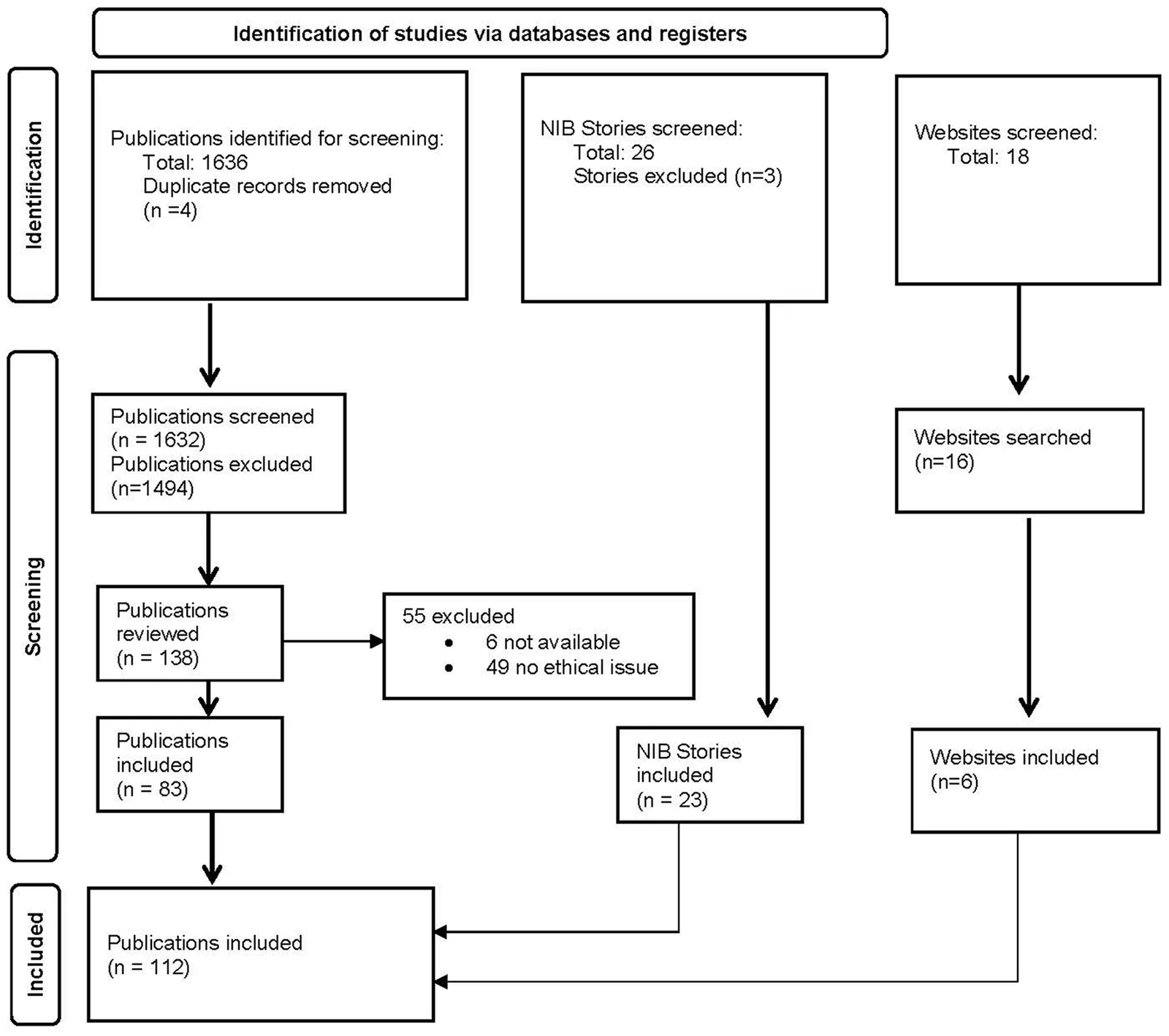
PRISMA diagram of systematic review inclusion and exclusion. 1636 publications, 26 published narratives and commentaries from a journal issue on medical interpreting, and 18 websites were screened for this systematic review with 112 items included for analysis.

The selection process (see Figure 1) followed the PRISMA reporting guidelines (40). Three authors (AI, JT, and SB) reviewed the titles and abstracts of the same 25 publications to refine inclusion/exclusion criteria and to improve inter-rater reliability. Two authors (JT and SB) completed title and abstract screening of all remaining publications, eliminating publications that clearly did not meet our inclusion criteria. Differences were resolved through discussion and in consultation with two other authors (AI and KM), allowing us to achieve unanimity. One author (AI) reviewed a selection of titles and abstracts to audit the process. We obtained full texts for all of the remaining publications. Three authors (AI, JT, and SB) conducted full text screening of the same 25 publications separately and compared results to foster interrater reliability and to refine the inclusion/exclusion criteria, an approach informed by recommendations for improving interrater and intercoder reliability in qualitative content analysis research (41, 42) and systematic reviews (43, 44). Two authors (JT and SB) conducted full text screening on all remaining publications. Discrepancies were resolved through discussion and, if necessary, in consultation with two other authors (AI and KM), allowing us to achieve 100% agreement. No additional publications were added based on the review of reference sections of included publications.

### Data extraction and synthesis

We created a data extraction form in Covidence, extracted data from the same ten publications, and compared results to refine the data extraction form, develop data extraction codes, and foster inter-rater reliability. Two authors (SB and JT) extracted data from the remaining publications. They consulted with each other and with two other authors (AI and KM) after screening 25 publications to compare results, refine the data extraction form, and resolve discrepancies through discussion, allowing us to achieve 100% agreement.

For each article, basic data on the background of the article was collected, as well as information about the ethical issues and interpreter roles discussed in the paper:

- Study ID
- Publication Information: full citation, title, lead author, year of publication, publication type
- Healthcare Setting
- Interpreted and Target Language
- Ethical Issue Identified: accuracy, neutrality, confidentiality, interprofessionalism, time constraints, professionalism, moral or emotional distress
- Ethical Issues: whether the ethical issues are explicitly recognized as ethical issues or they were implicit based on the judgment of authors of this review
- Interpreter Role: linguistic conduit, cultural broker, patient advocate
- Notes: qualitative summary of other relevant information

As recommended by Saldaña, we developed our codebook deductively and inductively (45). Prior to the study, we identified three main interpreter roles described in the literature: linguistic conduit, cultural broker, patient advocate (Table 1). The definitions of these roles that were used to extract data were informed by the National Codes of Ethics for Healthcare Interpreters (46) as well as various state guidelines for interpreters, previous systematic reviews concerning medical interpreters (33, 36). Based on our knowledge of the area, we began screening with a list of possible themes, and final codes were developed inductively. After reviewing the first set of 25 articles using qualitative content analysis and in consultation among all authors, we identified and defined six major categories of ethical issues (Table 2). We created a codebook and extracted ethical issues data using these terms. After we identified a seventh ethical issue category during discussion of our findings, we added “emotional or moral distress” to our codebook and reviewed all publications again to code for this seventh category. Tables 1, 2 report the number of publications that addressed these roles and ethical issues, respectively.

**Table 1:** Roles interpreters play.

Interpreter role	#	Definition
Linguistic conduit	101	A role whereby the interpreter acts as a transmitter of words from one language to another, without any omissions or additions; the interpreter here is a “pathway” for the words or meaning of an uttered or signed language to achieve the closest exact meaning in another language (46).
Cultural broker	58	A role whereby the interpreter facilitates cultural understanding by conveying cultural insights not known by the provider or the patient, usually to compensate for lack of knowledge on the part of the provider (46).
Patient advocate	53	A role whereby the interpreter elects to represent the needs and wishes of the patient to the provider team, especially when these needs and wishes are not explicitly stated by the patient (i.e., the interpreter is not directly communicating needs that the patient has articulated but instead has identified needs or interests that the interpreter believes should be advanced, represented, or protected) (46).

#: Number of publications that mention each role.

Medical interpreters have three main roles: linguistic conduit, cultural broker, and patient advocate (46, 52, 63, 109). Literature was reviewed to determine which (if any) of these roles were discussed and how many articles mentioned each role (#).

**Table 2:** Categories of ethical issues used for data extraction.

Ethical issue	#	Definitions	Examples of ethical decisions
Accuracy	74	The interpreter's ability or willingness to faithfully interpret spoken words from one language to another (46).	Deciding whether to directly translate a physician's statement when the statement is insensitive and the physician presumed the patient would not understand.
Neutrality	66	The adherence of the interpreter to an impartial attitude or stance in a medical encounter; the interpreter's ability or willingness to remain neutral even when given reason to move beyond the interpreting role (46).	Deciding whether to engage in conversation with patients while waiting for healthcare professionals or to remain silent and communicate only when actively interpreting.
Privacy and confidentiality	28	The interpreter's commitment to respect the privacy and confidentiality of all parties involved in the medical encounter (46).	Deciding whether to disclose to a healthcare professional that the interpreter has worked with the patient before or to disclose information the patient shared when the clinician was not present.
Inter-professionalism	60	The willingness and ability of healthcare professionals to include interpreters, and the ability of teams that include an interpreter to work cohesively to deliver effective patient care (46).	Deciding whether to agree to perform tasks healthcare professionals request that are outside the scope of an interpreter's responsibilities, such as calling patients independently to check on them or remind them of appointments.
Professionalism	43	The respect of professional and ethical norms and boundaries, and adherence to institutional standards (46).	Deciding whether to interpret in the care of patients undergoing procedures the interpreter finds morally objectionable, such as induced abortions.
Time constraints	25	Difficulties presented to an interpreter through a shortage of time for either the interpreter or provider team (76).	Deciding whether to resist a healthcare professional's attempt to save time by not allowing the interpreters to explain their role to give patients an accurate understanding of who they are and to build trust.
Emotional or moral distress	37	Difficulties and personal anxiety due to subjective feelings about a situation or concerns about doing the right thing, but finding institutional barriers that make it difficult or impossible to do so (85, 88).	Deciding what to do when they believe they ought to advocate for patients who are being denied information, care, or benefits to which they are entitled but doing so could result in termination of employment.

#: number of publications that mention each ethical issue.

Seven major categories of ethical issues were identified. Ethical issues arise when there is “a question of what one ought to do, rather than what is usually done or can be done” and answering the question requires “a resolution of value choices, as opposed to resolving merely factual or scientific matters” (110). Such questions emerge when there is tension among what various values, beliefs, principles, duties, rights, interests, or obligations permit, prohibit, or require us to do, or when there is uncertainty about what these factors require, permit, or prohibit. Concerns often involve decisions that may promote good, cause harm, threaten particular rights such as the right of competent adults to make their own healthcare decisions, affect justice or fairness, or impact personal or professional integrity (83, 110, 111). We defined each category and identified the number of publications that discussed them (#). Additional examples of each category appear in Results and Discussion below.

## Results and discussion

As depicted in Figure 1, the initial search of PubMed and MLA yielded 1636 publications for further analysis. These results were imported to Zotero for de-duplication. After removing 4 duplicates, these were imported to Covidence for screening. Title and abstract screening resulted in 1494 excluded publications. We sought to retrieve the full texts of the remaining 138 publications. Six were not available to us and had to be excluded. Full text screening led to exclusion of 49 publications because no ethical issues relating to a trained interpreter were identified. An issue of *Narrative Inquiry in Bioethics* on ethical issues faced by people who interpret for others in healthcare was added for screening after it was published in 2024. Of the 26 stories and commentaries in that issue, three were excluded because they were exclusively about *ad hoc* rather than trained interpreters. The remaining 23 were included. In addition, we identified 18 websites to review through the gray literature search. Two websites were excluded: one was inaccessible at the time of the search, and one did not have material on medical interpreters. Sixteen websites were included in the review (Table S4) and six entries made it to data extraction. This left us with a total of 112 publications (Table S5).

### Roles: ethical considerations

As described above, interpreters play three main roles, and they may adopt one or more of these at any given time. Our analysis found that 101 publications discussed the role of a linguistic conduit, 58 discussed the cultural broker role, and 53 discussed the patient advocate role (see Table 1). Tensions among these roles are documented here and elsewhere (20, 33, 47–51). These tensions can result in ethical questions and challenges for interpreters.

The most frequently mentioned role is the linguistic conduit. According to the California Healthcare Interpreting Association, “In this role, interpreters listen, observe body language, and convert the meaning of all messages from one language to another without unnecessary additions, deletions, or changes in meaning” (52). Most publications noted ethical concerns that arise because the linguistic conduit role is incompatible with the two other roles (cultural broker and patient advocate). In addition, the role might not advance the overall purpose of medical interpretation, which is to “facilitate accurate exchange of information, ensuring that both the physician's and the patient's voices are clearly heard and understood” (3). High quality patient-provider communication is critical for fulfilling ethical obligations, such as respecting patient autonomy, promoting good and equitable health outcomes, and advancing patient wellbeing (53–62). Strict adherence to the conduit role may make it difficult to fulfill these obligations, as the examples below illustrate.

The second role medical interpreters play is that of cultural brokers, which is important when cultural differences or nuances are relevant to provider-patient communication (63). For example, an interpreter who explains culture-specific information to a provider about cancer taboos acts as a cultural broker. A study that explored the importance of the broker role for advance care planning revealed many examples of cultural differences that make it difficult for patients to understand what they are being asked even if the words are communicated accurately because they are unfamiliar with the concepts (64). For example, in some cultures it is considered inappropriate, disrespectful, and even harmful to discuss death: “If I'm talking about death, I'm going to actually make it more likely to happen and therefore I'm being disrespectful of you” (64). Thus, a medical interpreter asked to interpret an advance care planning conversation may need to bridge the medical culture with the patient's culture for the language and concepts to be intelligible to the patient and to avoid offense, disrespect, or the perception that the patient is being harmed. In some cases, this cultural bridging may require that health professionals approach advance care planning differently for some patients.

The third role of medical interpreters is patient advocate. According to the California Interpreting Standards, in this role, “interpreters actively support change in the interest of patient health and wellbeing” (30). Another guideline states that “… the patient advocate role foresees interpreter action “outside the bounds of an interpreted interview”, which suggests that interpreters may have occasion to interact directly with patients in the absence of healthcare providers” (52). However, some practitioners argue that such interaction between patients and interpreters “violates professional distance and undermines the impartiality of the interpreter” (52). Interpreters may face situations in which they believe that a patient needs an advocate and must decide whether to intervene.

The plurality of interpreter roles gives rise to conflict or tension among them and, in turn, to ethical issues or questions. And, deciding which role to play and under what circumstances is ethical decision that and can affect the quality of patient care and their relationship with the healthcare team. Deviating from the conduit model can result in discipline or termination of the interpreter due to the healthcare team viewing this as the only appropriate role interpreters (49). Yet, providing direct interpretation can undermine effective and respectful communication. For instance, direct interpretation of some terms and concepts could be seen as disrespectful by patients and might not help them to understand the information either because there is no comparable term for their language (e.g., “goals of care”) (65) or because the concepts involved are not familiar in their culture (e.g., advance care planning) (64). “Commitment to accuracy or acting as a neutral conduit was perceived as uncomfortable when provider language was untranslatable or culturally inappropriate,” one publication stated (66).

Consider also a situation in which an interpreter notices that a patient is not asking questions and simply nods in agreement, but whose body language indicates a lack of understanding (67). An interpreter acting in a conduit role would remain silent. However, a cultural broker might reason that the patient is nodding in agreement because of cultural norms that call for deference to physicians and thus intervene to ensure that the patient receives a better explanation and is able to ask questions. Drawing attention to this cultural difference could be seen merely as explaining a cultural difference to a health professional for the patient's benefit, but some might describe such an intervention as a form of advocacy or as inappropriately challenging their authority or threatening their control over a patient encounter (33), which a healthcare professional might not welcome. This could result in restricting the interpreter's ability to advocate for or build relationships with patients. Strict adherence to the conduit role may also contribute to moral distress for interpreters.

Furthermore, determining which role is appropriate itself raises ethical issues in the literature, where we see that some people think that interpreters ought to play only one role and that moving to other roles is unethical, or at least unprofessional, while others disagree (64, 67). One question here is who should be in authority to determine what role is appropriate for an interpreter in a given situation. Interpreters have expressed frustration or concern because of lack of guidance about how to determine which role is appropriate (67). We found very little attention to patients' preferences regarding interpreter roles. Tension among the roles and different parties' perceptions of which roles interpreters should play and when are among the major contributors so the ethical issues that emerged in the literature, which we describe below.

A better understanding of the factors that give rise to the tension among roles and disagreements about which roles interpreters should play could help to identify solutions and ways to avoid some of the ethical issues discussed below as well as foster consistency in practice. All three roles support the goal of ensuring that all parties are “clearly heard and understood” and that the information exchanged is accurate (3). Yet, playing a role that advances one aspect of the goal, such as being understood, might be seen as undermining another aspect of the goal, namely being accurate. Tension among practices required to meet a compound goal (one with multiple elements) is not uncommon (68). Acknowledging that the purpose of medical interpretation involves a compound goal and that efforts to advance one aspect of that goal could be in tension with other aspects could allow for clarity about the nature of disagreement, thoughtful reflection on how to manage the tension, and a recognition that disagreement among parties about appropriate roles may be based at least in part in a concern for doing what each party considers is best for patients.

It is also possible that healthcare professionals who want interpreters to serve as linguistic conduits and only translate what they and patients say precisely believe that this is what will ensure the accurate exchange of information and is critical to understanding and being understood. They may be unaware that direct translation can undermine rather than advance their desired end. People who are not fluent or proficient in more than one language might not realize how often literal translations are inaccurate and lead to misunderstandings. Other possible explanations for conflicting views about the proper role of interpreters include fear of loss control and authority, lack of trust, and the severe time constraints healthcare professionals face (as described below).

### Categories of ethical issues

**Figure 2:**
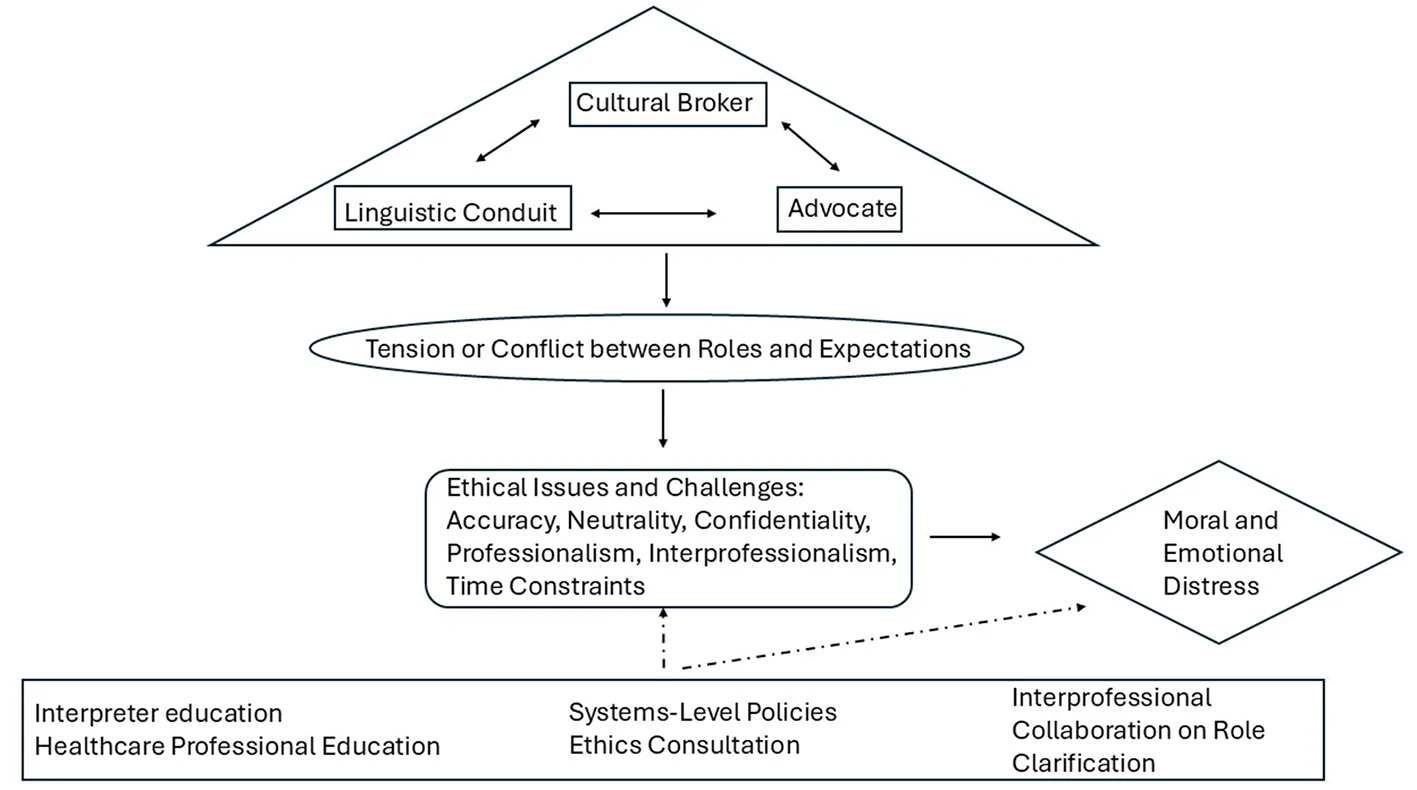
Plurality of roles medical interpreters play. Medical interpreters play three main roles – linguistic conduit, cultural broker and patient advocate. Tension among the roles and disagreements about which roles they should play contribute to ethical challenges in their work: accuracy, neutrality, confidentiality, interprofessionalism, professionalism and time constraints. Multiple approaches at the individual, professional, interprofessional, and systems level could alleviate some of these tensions and conflicts.

We identified and coded included publications for six categories of ethical concerns: accuracy, neutrality, confidentiality, interprofessionalism, professionalism and time constraints (see Table 2). Figure 2 provides a conceptual overview of three main roles interpreters play, the tensions among them, the six ethical issues identified in this review, many of which arise at least partially from those tensions, the relationship among them, and possible responses. One additional theme emerged during our discussion of findings across the categories of ethical issues and interpreter roles: interpreter emotional and moral distress. All publications were reviewed again to code for this seventh category after this cross-cutting theme emerged in our discussion of findings.

#### Accuracy

Accuracy, the ability of interpreters to closely interpret ideas and meaning from one language to another, was the most common ethical issue identified, with 74 publications mentioning it. Accuracy is closely linked to issues associated with trying to stay within the conduit role. For example, interpreters can face concerns when their commitment to complete accuracy could hamper communication with a patient, undermining the ethical obligation to facilitate understanding of information necessary to respect patient autonomy and to prevent harm caused by misunderstanding or lack of information. One author recounted an experience shared by a colleague who was interpreting for a patient receiving test results that revealed she was HIV-positive: “If she conveyed the physician's words exactly in the patient's language, my colleague was afraid that (the) patient would enter into a state of shock and would fail to take in the important and complex information about treatment options that the physician would no doubt give to her” (69).

When health professionals say something that they would not say in front of a patient who could understand them, they may not want those words communicated. The interpreter must decide whether or not to serve as a conduit and interpret the words accurately. An interpreter describes such a scenario: an elderly man with severe scoliosis meets a medical resident who saw the patient “and jumped up in excitement. With a great smile on his face, he exclaimed, “Wow! This is the worst case I have ever seen.” I felt startled. I could see that the patient was unsure about the resident's excitement. Normally, I would just go ahead and interpret what the physician said. I would then introduce the patient to the physician, explain my role as an interpreter.” However, this situation was different “… not because I was unsure about the linguistic equivalences of the resident's comment in Chinese. Rather, I was unsure how to best convey the information in a faithful, neutral, and accurate way without risking the provider patient relationship and the quality of care.” The interpreter chose to introduce herself and her role to the physician and “asked, ‘How would you like me to interpret what you just said? You appeared very excited.' The resident was taken aback, paused for a moment, and said, ‘Please tell him that I'm very happy to meet him.' I interpreted that and proceeded to my regular routines” (70). Deciding to ask the physician for clarification and to sacrifice accuracy to preserve the relationship and protect the patient from insensitive or offensive comments was an ethical decision. Interpreters need to be prepared to recognize and respond to challenging ethical situations, often without time to deliberate or consult with others. Some authors report that interpreters are rebuked for forgoing accuracy under any circumstances [e.g. (69)]. This is an example of how a mismatch between different understandings of the purpose and goal of interpreting can lead to tension or conflict.

#### Neutrality

Neutrality is regarded as necessary for facilitating communication within a healthcare setting, ensuring that interpreters respect professional boundaries, and protecting interpreters from emotional distress (46, 65). As one author described: “…interpreters are trained to avoid any verbal, physical, or emotional interactions with the patients without the presence of providers: they are only the voice of others” (49). Ethical questions regarding neutrality were often linked to decisions about staying within a conduit role vs. being an advocate or cultural broker.

Interpreters who identify with patients because of something they share in common, such as “race, ethnicity, country of origin, migration story, religion, and cultural norms” risk compromising their neutrality (64). When an interpreter and patient share a culture, there is a risk that the interpreter may feel a stronger connection or attempt to prevail upon a patient to adhere to a cultural norm as the interpreter understands it. Consider this example shared in an interview with a medical interpreter: “an interpreter, on two occasions, telling the wife….‘listen to your husband….You need to obey your husband because a good Hispanic, Christian woman obeys her husband!”' (67).

Situations in which interpreters communicate with patients beyond strictly interpreting, including conversations that happen without a health professional present, such as in waiting rooms, sometimes raise concerns. Whether these conversations risk neutrality and are problematic or can improve communication and trust is debated. Dysart-Gale notes: “some practitioners argue that to interact directly with patients in the absence of healthcare providers….violates professional distance and undermines the impartiality of the interpreter. Others argue that unmediated exchanges, such as chatting in the waiting room about the reasons for the patient's visit, can improve interpretation by orienting the interpreter to the patient's needs” (67). Another threat to neutrality and where we see tension among interpreter roles is when patients ask interpreters for guidance on services that might help them (71). An understanding of interpreters as advocates might call for interpreters to engage such requests while the conduit and cultural broker roles might treat these as outside the scope of their professional work.

Neutrality also requires that interpreters separate their personal opinions and cultural commitments from their work and withhold commentary on what they believe about healthcare encounters or decisions. One interpreter recounted this situation: “The doctor was telling a pregnant girl that she needed a test - an amniocentesis….and the interpreter said “oh, she's telling you get this test, but it's not a good one and you don't need it, so forget it” (67). Another author noted that sometimes “it is difficult to always tell what is culture-specific and what is personal opinion” (71). This can make it hard to discern whether neutrality has been violated.

#### Privacy and confidentiality

Respecting patients' privacy and confidentiality are well-recognized ethical and legal obligations in healthcare. Sometimes, this duty can raise ethical concerns for interpreters, which were mentioned in 28 publications. Interpreters sometimes have access to information or insights that they believe are relevant to patient care, but they cannot share them due to confidentiality, even though withholding information could be harmful to the patient. One author described the problem in the context of interpreting in the mental healthcare setting this way: “…the interpreter may witness that the patient is withholding the truth, but due to confidentiality they have to be like an “accomplice” which to many interpreters is ethically problematic” (72).

Concerns can arise when patients share information with interpreters outside of the presence of a healthcare professional. While walking between appointments, an interpreter learned from a patient that her husband hit her (73). The interpreter could not share this information with her healthcare providers without the patient's permission, and yet she believed it was important for her care. Eventually, the patient agreed that she could share this, which resulted in helping the patient to get additional assistance. Later the same day, the interpreter had to protect the patient's privacy in a different way, namely when the patient's husband came to the clinic and was looking for the patient. He bumped into the interpreter and started asking where he could find the patient, his wife. The interpreter claimed not to know her.

We found two ethical challenges related to confidentiality. One is whether to disclose to a provider having worked with a patient previously, which would breach patient confidentiality (14). It was interesting to see this concern noted; it underscores the fact that interpreters are not always considered and do not always see themselves as part of the healthcare team. For instance, it is routine for nurses to share information about a patient they have cared for with other nurses who are assuming care responsibilities. Second, some patients express concern about interpreters maintaining confidentiality (74, 75). This issue can be particularly significant when patients and interpreters come from the same community, as can happen among refugees in a particular region (75). Such concerns could lead to patients withholding information from healthcare professionals when interpreters are present, which can harm patient care.

#### Time constraints

Ethical issues associated with time constraints arose in 26 publications. Interpreters described feeling pressured to alter or diminish standard procedures because of time: “Interpreters were trained to provide information about their roles…at the beginning of each medical encounter. However, many interpreters complained about being under [pressure] to skip that introduction because providers do not have time to listen to them,” making it harder for patients and healthcare professionals to understand the interpreter's role (76). This can lead to other problems, such as patient fear of disclosing information in the presence of interpreters because interpreters have not been able to explain their commitment and obligation to protect privacy and confidentiality or to build trust.

Time constraints can impede the flow of information in a patient encounter, which can impact decision making: “During observations, it became clear that not only did important information have to be delivered within a short period of time, but important decisions had to be made before the professional interpreting time was up” (77). Patients who rely on interpreters might not receive as much information or might be pressured to make decisions more quickly than other patients. This inequitable treatment can adversely affect a patient's opportunity to obtain information and ask questions as is necessary to make informed decisions and adhere to medical advice, which can lead to worse health outcomes (12, 13, 29, 31, 56, 62).

Medical professionals sometimes choose not to request interpreter services out of concern that doing so will take additional time and disrupt busy clinic schedules (78). In other cases, such as in emergency situations, there might not be time to request services. Failure to request interpreter services denies patients access to support they need to facilitate communication, that leads to better outcomes, and to which they have a legal right in the U.S. and many other countries.

Another way in which time constraints can adversely affect the quality of interpretation or the experience of interpreters is the failure of healthcare professionals to brief interpreters before patient encounters, which can “help support them emotionally and also enhance their ability to translate appropriately” (79). This can be especially important for complex clinical encounters, such as when delivering a serious diagnosis, a significant change in prognosis, or broaching end of life decisions. One author described positive changes in the interpreting experience after the team implemented a “pre-huddle” prior to patient appointments during which providers would quickly “brief the interpreter about the patient's medical condition and the goal of treatment… (and) solicit any input from the interpreter around language and cultural challenges” (80).

In addition to the reality that healthcare professionals often are extremely pressed for time, it is possible that some of the difficulties interpreters describe with respect to explaining their role to patients or meeting with clinicians before an encounter to learn about the patient's situation are rooted in an issue discussed above, namely disagreement about an interpreter's role. A health professional who believes interpreters should only play the role of conduit likely sees no value in allowing the interpreter to communicate with the patient directly or to interact with the team other than directly translating their words.

Discussions about time constraints affected interpreters' professionalism and interprofessional relations, the next two areas we consider.

#### Professionalism

The weighty nature of medical interpreting demands a high standard of professionalism which can place unique pressures on interpreters. Forty-four publications mentioned ethical concerns associated with professionalism. Sometimes, interpreters may be part of interactions that involve services or decisions that they find morally objectionable, such as induced abortions (81). Interpreters' professional obligations “may require them to provide their services despite the matter at hand being in conflict with their personal (moral) beliefs when, for example, they are the only individuals available to effectively facilitate communication, and the consequences of not doing so would be significantly negative” (81). For instance, an interpreter could be asked to help with healthcare interventions they find morally problematic.

Emotional or affective dimensions of healthcare interpretation can undermine an interpreter's professionalism. One interpreter offered this example: “Sometimes you soften things. I had a case where we walked in, [the] doctors said, ‘This is your scan. You've got a huge tumor on your kidney. There's nothing I can do about it.' I could not do that” (66). In “softening things,” the interpreter did not convey information accurately and completely, which has a potential for harm and may be seen as a violation of professional obligations. At the same time, the interpreter might believe that interpreting directly when a healthcare provider has been too blunt could harm a patient.

Bilingual healthcare professionals who are trained to work as interpreters may face tension between the professional norms of interpreting and those of their healthcare profession, which they might have to set aside in their interpreting work. The codes of ethics for interpreters call deference to healthcare professionals. In a way, they are asked to be rather passive and not to build relationships with patients (49). In contrast, the codes and norms for other health professions typically emphasize building relationships and trust with patients and ensuring that patients receive quality care, yet interpreters are expected to be somewhat invisible (49). Role tensions also are present in the case of student interpreters, as they have to juggle their goals of learning with the demands of interpreting in a patient interaction (82). Someone who prioritized the opportunity to learn more about the medical aspects of a high stakes or complex case by, for example, asking the clinician questions for the sake of knowledge-building rather than interpreting, might shortchange the communication between patient and provider, undermining the quality of interpretation.

#### Interprofessionalism

Medical interpreters always work alongside a team of health professionals. Thus, it is not surprising that over half of the publications (*n* = 60) mentioned ethical issues associated with interprofessional relationships. Situations often arise where physicians and interpreters do not coordinate their communication with the patient. As noted in the discussion of time constraints, several publications mentioned the importance of having a meeting between the interpreter and health professional prior to an interpreting encounter when the situation is complex (62, 83). Such discussions may help to improve the quality of interpretation and prevent some of the emotional and moral distress interpreters experience, as discussed below.

As difficult as it is for patients not to understand the language in which a health professional is communicating, health professionals also may be concerned that patients and interpreters are communicating in ways that they do not understand and that they may be missing important information. For example, one physician interviewed about experiences of working with interpreters said, “Some [interpreters] talk and talk [with patients] and then turn to me and say, “She said yes.” I need to know what I'm missing” (67). Furthermore, although interpreter services receive positive appraisals, many physicians report concerns over the quality of interpretation. One study reported that “Physicians' concerns ranged from inaccurate interpretation, incomplete information to suspecting that the interpreter does not endorse their communicative agenda…” (82).

Health professionals sometimes ask interpreters to take on responsibilities that are beyond their scope of practice or duties. Examples include staff asking interpreters to provide patients with all discharge instructions or call patients to check on them or remind them of upcoming appointments (67). These requests pose ethical concerns for interpreters in part because they might be asked to do things they are not qualified to do and in part because these demands can take time away from their assigned responsibilities. At the same time, failing to fulfill these requests could further undermine patients' interests because they will be left without needed assistance or instructions.

Some publications we reviewed pointed to physicians and other medical staff not being properly trained on how to interact with interpreters or being unaware of the various roles an interpreter can play (35). One paper on interpreting in the speech pathology context described such confusion as common: “Unclear role expectations for speech pathologist and interpreter participants were also reported as both a common actual and potential challenge. Speech pathologist participants were purportedly unsure about the interpreting process and therefore had unrealistically high expectations of interpreter participants' skills and abilities…Interpreter participants were also unclear about their roles in speech pathology sessions and were unsure of when to interpret information during the sessions…” (84). This uncertainty interfered with the ability of interpreters to fulfill their professional obligations on time (85). Miscommunication or lack of understanding about the interpreter's role in relation to the health professional can lead interpreters to be uncomfortable fulfilling their roles as advocates or cultural brokers. Or, when medical teams do not recognize these roles as legitimate or respect them and instead assume that interpreters will or should serve only as conduits, interpreters might be unable to effectively perform their full range of duties.

Although many papers described ethical concerns associated with interprofessionalism, not all of the discussions of interprofessional relationships in the publications were negative. Several described positive examples of teams that included interpreters, demonstrating that strong interprofessional relationships can foster ethical practice. One interpreter described a positive experience in a labor and delivery unit:

“I was present on the day of delivery. As I interpreted the emotional complexity of this event, a deep sense of empathy washed over me. I realized I could serve by giving my love through my words, my silence, and my presence. I interpreted for the mom when there were English-speaking care providers, focusing on interpreting for the patient's mom and spouse when needed. I was quiet in the times when a Spanish-speaking care provider spoke to the patient and family. ….In an act of solidarity, I approached her, touching her head and hands to give her strength and comfort at a challenging time. I followed the exceptional leadership of the nurses, who demonstrated steadfast compassion and professionalism. Working with a medical team that recognizes the interpreter as a professional team member and is open to collaboration can enhance the quality of healthcare interactions. We serve as a vital link between the medical team and, in this case, the expectant mother, facilitating clear communication and understanding.” (86).

This account also underscores the importance of attempting to build rapport with the patient rather than being a detached language conduit, a topic noted above in the professionalism discussion.

### Emotional and moral distress

In discussing our findings, we noted that many of the ethical issues that are associated with the three interpreter roles and the six categories of ethical issues discussed above contributed to emotional or moral distress for interpreters. Emotional distress compasses “subjective feelings that vary in intensity from sadness, uncertainty, confusion and worry to more significant symptoms such as anxiety, the expression of anger, social isolation and hopelessness” (85). One source of emotional distress we saw across publications was strict adherence to the conduit role. For example, Hsieh found that interpreters report distress because they often were not permitted to advocate for or comfort patients and were expected to act only as language conduits. Some faced severe consequences for expanding beyond this role: “Interpreters' failure to follow a conduit model often is viewed as a transgression that may warrant termination of their jobs” (87). The view that interpreters should serve as conduits only, that professionalism requires neutrality, and that interpreters should not attempt to build relationships with patients can leave them feeling isolated and emotionally disconnected (87).

Moral distress, a concept that emerged in the nursing literature, refers to situations “when one knows the right thing to do, but institutional constraints make it nearly impossible to pursue the right course of action” (88). The concept has expanded over time and there is now a broad literature on moral distress across the health professions (89–92). We found examples of moral distress in many publications. These include witnessing systemic issues that pose serious problems for patients and interpreters, such as failures to follow laws and policies meant to facilitate access to interpreters, use of healthcare professionals' own limited language skills to communicate with patients and avoid using interpreters, and reliance on online translation tools rather than interpreters [see, e.g, (93)]. Without effective policies and practices to address these problems, moral distress can build. Interpreters also may experience distress when they interpret for patients undergoing specific procedures, such as induced abortions, or having experiences that hit close to home for the interpreter, such as pregnancy loss (94). Navigating these situations and finding effective ways to maintain professional boundaries and provide appropriate interpreting services while minimizing their own distress can be difficult.

Our findings demonstrate how different ethical concerns can contribute to additional ethical challenges, which compounds the difficulties patients, clinicians, and interpreters face in complex healthcare landscapes.

One limitation of this study is that we reviewed only on publications in English, possibly missing themes that arose only in publications written in other languages. It also means that our sample is skewed toward healthcare interpreting in English speaking contexts, though several included publications were from countries where English is not the primary language, such as Denmark, Sweden, and Singapore (72, 95, 96). Conducting similar mapping reviews in other languages could facilitate a comparison. A second limitation is that we used only two databases. Although both have very wide reach and the decision to focus on these two was based on preliminary searches of additional databases which did not yield additional relevant publications, it is possible that we missed literature not included in either of those. Third, we conducted this review at a time of rapid change regarding deployment of artificial intelligence tools in healthcare settings. The implications of this for interpreting remain unclear, and the literature is limited thus far. It is likely that such tools will be used to replace trained interpreters in many settings, and this will raise significant ethical questions and merits careful study.

Finally, as is typical for mapping reviews, we did not perform quality assessment of the studies included (97). Restricting ourselves to the kinds of publications where quality assessment would have been appropriate would have severely limited our research because much of the work available is not from research studies.

Our review included different types of publications, including peer reviewed journal articles (systematic reviews, articles reporting qualitative and quantitative experimental research results, and conceptual papers, case reports/series), personal narratives, commentaries, and websites. Traditional evidence hierarchies in healthcare treat these differently, with systematic reviews, metanalyses, and randomized controlled trials occupying the top tiers of pyramid (98, 99). The hierarchy reflects that the confidence we may have in what a source of evidence shows and the conclusions we may justifiably draw from it, that is, “the relative certainty of evidence, namely the power to make inferences,” varies [(100), p. 3]. We cannot draw from a collection of case reports or personal narratives, for example, that the majority of interpreters have experienced a particular ethical challenge. Nor can we establish causal connections between particular environments and ethical concerns. However, there is growing recognition of the importance of using a range of sources of evidence to better understand real world evidence and use it to inform practice (100, 101). We relied on a wide variety of publication types to maximize identification of ethical issues and challenges. Because many of the publications would not meet the criteria for inclusion in a systematic review, failure to include diverse publication types would not have accounted for some of the ethical issues and challenges that emerged in our analysis. Publications that share first-person experiences and expert insights provide a window into lived experiences that quantitative measures can miss. At the same time, because of the heterogeneity of our sources, we cannot draw answer certain types of questions that are important. This mapping review raises many questions that cannot be answered with the available evidence and that warrant further study. This includes questions about whether the kinds of ethical challenges that arise vary by healthcare setting, such as acute care vs. long-term care or outpatient primary care, or type of care healthcare professional, for example, physician vs. nurse. We draw tentative conclusions below regarding possible ways to mitigate the ethical concerns identified, though these, also, require further investigation.

## Conclusion

In this study, we describe the three ethical roles (linguistic conduit, cultural broker, and patient advocate) of an interpreter and identified seven categories of ethical issues (accuracy, neutrality, confidentiality, professionalism, interprofessionalism, time constraints, and emotional or moral distress). We found that tension regarding roles and role conflict as well as the variety of ethical issues we identified sometimes resulted in emotional or moral distress. Our findings expose the complex ethical challenges that medical interpreters face in conducting their work and point to possible ways to address these concerns.

Some of these concerns could be alleviated by training medical professionals to ensure they understand the value of interpreters' different roles, the importance of providing patients with access to trained interpreters, and how to work and communicate with interpreters overall. As one author observed, “Education of health professionals in using a professional interpreter, and the professional interpreter's role in various care situations” is important for improving patient care (78). Several training programs exist for medical staff and students who would like to serve as interpreters (102). However, training should be provided for health professionals who use (or should use) medical interpreters to improve their understanding of value, importance, and roles of medical interpreters and to help them understand best practices for providing care to patients when there is a mismatch between patient and provider language (34).

Healthcare interpreters could benefit from additional training on working in particular contexts, such as mental healthcare, where information and relationships might be especially sensitive. Better education and training on how to identify and navigate ethical issues could be valuable as well. These efforts could draw from established methods for education health professions students and for providing ongoing education to health professionals, such as simulations and role plays, seminars, and didactic experiences (103–107). One challenge to promoting more ethics training for interpreters is that there is no standard training required of all interpreters, and people enter the practice through different paths. Implementing clearer standards and requirements could lead to some improvements, though the benefits would have to be considered in light of possible harms, such as increased costs and difficulty in finding qualified interpreters for some languages.

At the institutional or systems level, efforts to address ethical issues and reduce moral and emotional distress include ethics consultation services as well as guidance both from professional societies and institutions on the appropriate roles and responsibilities of interpreters and on how to facilitate patient access to interpreters as well as how to report and resolve barriers. Healthcare organizations could develop clearer policies on when interpreters should be included and improve staff awareness and understanding of those policies. These improvements could build on existing strategies already in place, such as hospital language access services, formal certification pathways, and interprofessional training services. However, our findings suggest that while these frameworks provide a foundation, more targeted and context specific guidance is needed to help clinicians and interpreters navigate ethically complex situations. Given the tension among different perspectives regarding the appropriate roles for interpreters, it is possible that an interprofessional effort to develop guidance on their roles and on how they should be incorporated into healthcare teams could promote improved collaboration. This could inform training for both health professionals and interpreters and lead to better experiences for all affected parties, including patients.

The literature thus far has paid little attention to the ethical issues that arise when trained interpreters are involved in patient encounters. This mapping review provides an account of three primary roles that medical interpreters play, the tension among these, and the types of ethical issues that emerge in this work. More empirical research to determine the full range and frequency of ethical issues and their impact as well as research that assesses ways to manage and mitigate these concerns is important. Healthcare institutions and professionals in the United States and other countries with significant immigrant populations likely will have more and more incidents where a medical interpreter is needed. In addition, when healthcare costs are under constant scrutiny and technological solutions such as AI tools are being explored as alternatives to human interpreters, it is critical to remember that interpreters bring a human dimension that facilitates navigating complex emotional dynamics, understanding nuanced references, and advocating in ethically fraught situations. Machines cannot replicate this at this time. Furthermore, these tools likely will raise many of the ethical issues that use of other AI tools in healthcare raises, such as concerns about patient privacy and confidentiality, accuracy, bias, and accountability and responsibility for errors (108). Assessing the use of these tools and their impact on patient care and health outcomes will be important in the future. Healthcare organizations implementing such tools should develop an assessment plan and evaluate the impact of their decision on patients and clinicians. Additional areas for future research include determining the ideal training for interpreters and the preferences of patients with respect to different approaches to healthcare interpretation as well as studying the experiences of patients, clinicians, and interpreters in low- and middle-income countries.
