# Supplementary material for: Interpreting in healthcare: a systematic search and mapping of ethical issues in health literature
Source: Front Public Health. 2026 Aug 18;14:1856115. doi: 10.3389/fpubh.2026.1856115 (PMC13531408; doi:10.3389/fpubh.2026.1856115)
Supplement: Supplementary file 1 [file Supplementary_file_1.docx]

**Appendix S1: Previous Literature Reviews on Health Care Interpreting**

**Table S1: Summary of Previous Reviews on Healthcare Interpreters**

| **Citation** | **Research Question(s)** | **Databases, Dates, # Included, and Key Findings** |
| --- | --- | --- |
| Bauer A.M., Alegría M. Impact of patient language proficiency and interpreter service use on the quality of psychiatric care: a systematic review. Psychiatric Services. (2010). (8):765-73. doi: 10.1176/ps.2010.61.8.765. [16] | What is the effect “patients limited English proficiency and use of professional and ad hoc interpreters on the quality of psychiatric care”? [15, p. 765] | PubMed, PsycINFO, and CINAHL. Dates: From database inception to April 2009. 26 included publications. --“Evaluation in a patient’s nonprimary language can lead to incomplete or distorted mental status assessment”  -“untrained interpreters’ errors may have greater clinical impact”  -“Use of professional interpreters may improve disclosure in patient-provider communications, referral to specialty care, and patient satisfaction” [16, p. 765] |
| Heath M, Hvass A.M.F., Wejse C.M. Interpreter services and effect on healthcare - a systematic review of the impact of different types of interpreters on patient outcome. Journal of Migrant Health. (2023). 7:100162. doi: 10.1016/j.jmh.2023.100162. [17] | What is the effect of the “use of interpreters in the medical setting … on quality of care”? [17, p. 1] | PubMed and Embase. No date limits. Dates: Final search conducted April 2021. 29 publications.  -Use of “in-personal professional interpreter result[ed] in highest satisfaction and communication.”  -“any interpreter is better than none and relational interpreters can be a valuable ….resource for patients in the private practice setting” [17, p. 1] |
| Boylen S, Cherian S, Gill F.J., Leslie G.D., Wilson S. Impact of professional interpreters on outcomes for hospitalized children from migrant and refugee families with limited English proficiency. JBI Evidence Synthesis. (2020). (7). doi: 10.11124/jbisrir-d-19-00300. [18] | “Does the use of a professional interpreter for LEP migrant and refugee families impact on:  -Child and/or parent satisfaction with care  in hospital?  -Children’s hospital length of stay, unplanned  readmission rates and non-attendance rates at  clinic appointments?  -Adherence to treatment, medication errors and  adverse events relating to the child in hospital?” [18, p. 1367] | MEDLINE, PubMed, ProQuest Central, Scopus, Web of Science, Embase, PsychINFO, Science Direct, APAIS Health AIHW, AustHealth, Factiva, Multicultural Australia and Immigration studies, and CINAHL Plus. Dates: Final search conducted December 2018. 7 publications.  -“Using professional in-person interpreters resulted in a shorter total emergency department throughput time compared to using professional interpreters via telephone”  -“no difference in concordance of child’s discharge diagnoses between …. Professional interpreters and ... bilingual physicians”  -“Video interpretation provided better understanding of diagnoses than phone interpretation” [18. 1360] |
| Flores G. The Impact of Medical Interpreter Services on the Quality of Health Care: a Systematic Review. Medical Care Research and Review. (2005). J62(3):255–99. doi: 10.1177/1077558705275416. [27] | What is “the impact of interpret services on quality of care?” [27, p. 255] | MEDLINE, CANCERLIT, CINAHL, HealthSTAR, PsycINFO. Dates: Searches conducted between October 2002 and January 2003. 36 publications.  -“quality of care is compromised when LEP patients need but do not get interpreters”  -“LEP patients’ quality of care is inferior”  -“more interpreter errors occur with untrained ad hock interpreters”  -“Inadequate interpreter services can have serious consequences for patients with mental health disorders”  -“trained professional interpreters and bilingual health care providers positively affect LEP patients’ satisfaction, quality of care, and outcomes” [27, p. 255] |
| Karliner L.S., Jacobs E.A., Chen A.H., Mutha S. Do professional interpreters improve clinical care for patients with limited English proficiency? A systematic review of the literature. Health Services Research. (2007).42(2):727-54. doi: 10.1111/j.1475-6773.2006.00629.x.  [28] | Do “professional interpreters have a positive impact on clinical care for limited English proficiency (LEP) patients”? [28, p. 727] | PubMed and PychINFO. Dates: 1966-September 2005. 28 publications.  -use of professional interpreters is associated with improved clinical care more than is the use of ad hoc interpreters” as measured by evaluating “communication (errors and comprehension), utilization, clinical outcomes, or satisfaction”  -“professional interpreters appear to raise the quality of clinical care for LEP patients to approach that for patients without language barriers” [28, p. 727] |
| Kwan M, Jeemi Z, Norman R, Dantas J.A.R. Professional Interpreter Services and the Impact on Hospital Care Outcomes: An Integrative Review of Literature. International Journal of Environmental Research and Public Health. (2023). 20(6):5165. doi: 10.3390/ijerph20065165. [29] | What is “the impact of professional interpreter services on hospital care outcomes and the associated cost of service provision”? [29, p. 1] | EBSCO, MEDLINE, ProQuest, PubMed, Scopus. Dates: January 1996-December 2020. 27 publications.  -“provision of professional interpreter services can enhance hospital care for linguistically diverse patients by improving patient-provider communication” [29, p. 1] |
| Silva M.D., Genoff M, Zaballa A, Jewell S, Stabler S, Gany F.M., Diamond LC. Interpreting at the End of Life: A Systematic Review of the Impact of Interpreters on the Delivery of Palliative Care Services to Cancer Patients With Limited English Proficiency. Journal of Pain Symptom Management. (2016). 51(3):569-80. doi: 10.1016/j.jpainsymman.2015.10.011.  [30] | How do “professional medical interpreters influence the delivery of palliative care services to LEP patients”? [30, p. 569] | PubMed, PsycINFO, Web of Science, Cochrane, Embase, Scopus. Dates: 1960 or 1966, depending on database-January 2013. 10 publications.  -“the quality of care provided to LEP patients receiving palliative care services is influenced by the type of interpreter used.”  - “When professional interpreters were not used, LEP patients and families had inadequate understanding about diagnosis and prognosis during goals of care conversations and patients had worse symptom management at the end of life, including pain and anxiety” [30, p. 567] |
| Wiles I, Tariq A, Karasneh G, Summerville D.J., Tovar D, Woltenberg L. The Effects of Interpreter Utilization on Patient Outcomes: A Contemporary Literature Review. Education in the Health Professions. (2023). 6(2):49. doi: 10.4103/EHP.EHP_4_23.  [31] | What are “the effects of interpreter utilization on clinician outcomes, such as length of stay, readmission rates, and patient satisfaction, in patients in the emergency department or inpatient hospital who have limited English proficiency (LEP)”? [31, p. 49] | PubMed. Dates: 1994-2021. 17 publications.  -“use of interpreters is positively related to outcomes in hospitalized patients with LEP in most outcome measures,” especially “patient satisfaction” [31, p. 49] |
| Vange S.S., Nielsen M.R., Michaëlis C, Smith Jervelund S. Interpreter services for immigrants in European healthcare systems: a systematic review of access barriers and facilitators. Scandinavian Journal of Public Health. (2024). 52(7):893-906. doi: 10.1177/14034948231179279[32] | What are “immigrants’ and healthcare professionals’ experiences with barriers and facilitators of interpreter services” And, what is “the influence of barriers to interpreter services on the quality of care from immigrant and healthcare professionals’ perspectives”? [32, p. 893] | PubMed, Embase, Cochrane Library, Scopus, SocINDEX, PsycINFO. 21 publications. Dates: 1996-2021.  -“barriers included: lack of immediately available interpreter services, cost, lack of knowledge about availability, and attitude towards interpreter services”  -“Facilitators included: a high number of interpreters in the requested language, awareness among healthcare professionals and patients of the patient’s rights to interpreters, and a positive attitude towards use of interpreter services.”  -“language barriers created safety risks for the patients, made patients feel unsafe, or delayed patient contact with the healthcare system.”  -*“*Medical encounters with unaddressed language barriers can put patients at risk and reduce quality of care for immigrants, which calls for strengthening formal and informal access to interpreters” [32] |
| Brisset C, Leanza Y, Laforest K. Working with interpreters in health care: a systematic review and meta-ethnography of qualitative studies. Patient Education and Counseling. (2013). 91(2):131-40. doi: 10.1016/j.pec.2012.11.008. [33] | What “relational issues [are] involved in working with interpreters in healthcare settings”? [33, p. 131] | PsycINFO, EBSCO Medline, Current Contents, Web of Science, CSA Linguistics, Language Behavior Abstracts, SCA Sociological Abstracts, Cochrane Library. Dates: database inception – June 2010. 61 publications.  -“Interpreters fill a wide variety of roles….This diversity and oscillation are sources of both tension and relational opportunities.”  - “The difficulties encountered by practitioners, interpreters and patients are related to issues of trust, control and power.”  -“Non-literal translation appears to be a prerequisite for effective and accurate communication” [33, p. 131] |
| Fukui N, Partain D.K., Yeow M.E., Farfour H.N., Prokop L, Barwise A. Learning to collaborate with medical interpreters in health professions educati: A systematic review of training programs. Medical Teaching. (2024). 46(2):258-272. doi: 10.1080/0142159X.2023.2249211.  [34] | What is “the effectiveness of existing training programs that teach optimal collaboration practices between clinicians and interpreters during patient encounters? [34, p. 258] | MEDLINE, Embase, Scopus, and Cochrane Central. Dates: 1945-June 2022. 19 publications.  -there is significant variation among training programs.  ‘”There is a critical need for structured programs to train clinicians to effectively collaborate  with medical interpreters to reduce healthcare disparities”  -“An effective training program should involve interpreters in the development and delivery of the program, practical skills development through interactive activities, structured clinical skill assessment, and both in-person and virtual components” [34 p. 258] |
| Sleptsova M, Hofer G, Morina N, Langewitz W. The role of the health care interpreter in a clinical setting--a narrative review. Journal of Community Health Nursing. (2014). 31(3):167-84. doi: 10.1080/07370016.2014.926682. [35] | What are the “published models of health care interpretation and associated roles, expectations, and outcomes”? [35]. | PubMed,CINAHL, PsycINFO, PSYNDEXPlus. Dates: not reported. 34 publications.  -“only 2 recommend strict adherence to the conduit model”  -“32 studies defined [interpreters’ roles] in broader terms as the role of a cultural broker (n = 18), a manager or clarifier (n = 22), a patient advocate (n = 13), ora mediator (n = 6)”  -“There are no commonly accepted understandings of the interpreters' role” [35, p. 167”] |

| **S2 Appendix: PRISMA Checklist** | | |  |
| --- | --- | --- | --- |
| **TITLE** | 1 | Identify the report as a systematic review. | Title (p. 1), Abstract (p. 2) |
| **ABSTRACT** | | |  |
| Abstract | 2 | See the PRISMA 2020 for Abstracts checklist. | Abstract (p.2) |
| **INTRODUCTION** | | |  |
| Rationale | 3 | Describe the rationale for the review in the context of existing knowledge. | Introduction (pp. 3-4) |
| Objectives | 4 | Provide an explicit statement of the objective(s) or question(s) the review addresses. | Introduction (pp. 3-4) |
| **METHODS** | | |  |
| Eligibility criteria | 5 | Specify the inclusion and exclusion criteria for the review and how studies were grouped for the syntheses. | Methods Search Criteria (pp. 5-6) |
| Information sources | 6 | Specify all databases, registers, websites, organisations, reference lists and other sources searched or consulted to identify studies. Specify the date when each source was last searched or consulted. | Methods - Search Criteria (p. 5) and Appendices S2 and S3 |
| Search strategy | 7 | Present the full search strategies for all databases, registers and websites, including any filters and limits used. | Appendices (Appendices S2 and S3) |
| Selection process | 8 | Specify the methods used to decide whether a study met the inclusion criteria of the review, including how many reviewers screened each record and each report retrieved, whether they worked independently, and if applicable, details of automation tools used in the process. | Methods (pp. 6-7) |
| Data collection process | 9 | Specify the methods used to collect data from reports, including how many reviewers collected data from each report, whether they worked independently, any processes for obtaining or confirming data from study investigators, and if applicable, details of automation tools used in the process. | Methods-Data Extraction and Synthesis (pp. 8-9) |
| Data items | 10a | List and define all outcomes for which data were sought. Specify whether all results that were compatible with each outcome domain in each study were sought (e.g. for all measures, time points, analyses), and if not, the methods used to decide which results to collect. | Methods – Data Extraction and Synthesis (pp. 8-10) |
|  | 10b | List and define all other variables for which data were sought (e.g. participant and intervention characteristics, funding sources). Describe any assumptions made about any missing or unclear information. | N/A |
| Study risk of bias assessment | 11 | Specify the methods used to assess risk of bias in the included studies, including details of the tool(s) used, how many reviewers assessed each study and whether they worked independently, and if applicable, details of automation tools used in the process. | N/A |
| Effect measures | 12 | Specify for each outcome the effect measure(s) (e.g. risk ratio, mean difference) used in the synthesis or presentation of results. | N/A |
| Synthesis methods | 13a | Describe the processes used to decide which studies were eligible for each synthesis (e.g. tabulating the study intervention characteristics and comparing against the planned groups for each synthesis (item #5)). | Methods – Data Extraction and Synthesis (pp. 8-10) |
|  | 13b | Describe any methods required to prepare the data for presentation or synthesis, such as handling of missing summary statistics, or data conversions. | N/A |
|  | 13c | Describe any methods used to tabulate or visually display results of individual studies and syntheses. | N/A |
|  | 13d | Describe any methods used to synthesize results and provide a rationale for the choice(s). If meta-analysis was performed, describe the model(s), method(s) to identify the presence and extent of statistical heterogeneity, and software package(s) used. | Methods - Data Extract and Synthesis (pp. 8-10) |
|  | 13e | Describe any methods used to explore possible causes of heterogeneity among study results (e.g. subgroup analysis, meta-regression). | N/A |
|  | 13f | Describe any sensitivity analyses conducted to assess robustness of the synthesized results. | N/A |
| Reporting bias assessment | 14 | Describe any methods used to assess risk of bias due to missing results in a synthesis (arising from reporting biases). | N/A |
| Certainty assessment | 15 | Describe any methods used to assess certainty (or confidence) in the body of evidence for an outcome. | N/A |
| **RESULTS** | | |  |
| Study selection | 16a | Describe the results of the search and selection process, from the number of records identified in the search to the number of studies included in the review, ideally using a flow diagram. | Results and Discussion (pp. 10-11) and PRISMA Flow Diagram Figure 1 (p. 7) |
|  | 16b | Cite studies that might appear to meet the inclusion criteria, but which were excluded, and explain why they were excluded. | N/A |
| Study characteristics | 17 | Cite each included study and present its characteristics. | Table S4 (Appendix S4, p. S4-2)) |
| Risk of bias in studies | 18 | Present assessments of risk of bias for each included study. | N/A |
| Results of individual studies | 19 | For all outcomes, present, for each study: (a) summary statistics for each group (where appropriate) and (b) an effect estimate and its precision (e.g. confidence/credible interval), ideally using structured tables or plots. | N/A |
| Results of syntheses | 20a | For each synthesis, briefly summarise the characteristics and risk of bias among contributing studies. | N/A |
|  | 20b | Present results of all statistical syntheses conducted. If meta-analysis was done, present for each the summary estimate and its precision (e.g. confidence/credible interval) and measures of statistical heterogeneity. If comparing groups, describe the direction of the effect. | N/A |
|  | 20c | Present results of all investigations of possible causes of heterogeneity among study results. | N/A |
|  | 20d | Present results of all sensitivity analyses conducted to assess the robustness of the synthesized results. | N/A |
| Reporting biases | 21 | Present assessments of risk of bias due to missing results (arising from reporting biases) for each synthesis assessed. | N/A |
| Certainty of evidence | 22 | Present assessments of certainty (or confidence) in the body of evidence for each outcome assessed. | N/A |
| **DISCUSSION** | | |  |
| Discussion | 23a | Provide a general interpretation of the results in the context of other evidence. | Results and Discussion (pp. 10-26) |
|  | 23b | Discuss any limitations of the evidence included in the review. | Results and Discussion (p. 26) |
|  | 23c | Discuss any limitations of the review processes used. | Results and Discussion (p. 26). |
|  | 23d | Discuss implications of the results for practice, policy, and future research. | Conclusion (pp. 27-28) |
| **OTHER INFORMATION** | | |  |
| Registration and protocol | 24a | Provide registration information for the review, including register name and registration number, or state that the review was not registered. | Methods (p. 5) |
|  | 24b | Indicate where the review protocol can be accessed, or state that a protocol was not prepared. | Methods (p. 5) |
|  | 24c | Describe and explain any amendments to information provided at registration or in the protocol. | N/A |
| Support | 25 | Describe sources of financial or non-financial support for the review, and the role of the funders or sponsors in the review. | Acknowledgements (p. 29) |
| Competing interests | 26 | Declare any competing interests of review authors. | Journal declaration page and p. 29 |
| Availability of data, code and other materials | 27 | Report which of the following are publicly available and where they can be found: template data collection forms; data extracted from included studies; data used for all analyses; analytic code; any other materials used in the review. | Data sharing section of ms. (p. 29). |

*From:*  Page MJ, McKenzie JE, Bossuyt PM, Boutron I, Hoffmann TC, Mulrow CD, et al. The PRISMA 2020 statement: an updated guideline for reporting systematic reviews. BMJ 2021;372:n71. doi: 10.1136/bmj.n71. This work is licensed under CC BY 4.0. To view a copy of this license, visit <https://creativecommons.org/licenses/by/4.0/>

# **S3 Appendix: Literature Search Strategy**

In consultation with a librarian from the Z Smith Reynolds Library at Wake Forest University, Colleen Foy, MLIS, we designed a search strategy. The strategy includes databases: PubMed, and MLA International Bibliography. The search strategy for each database is below. No date limits were used. Language limits were applied to limit publications in English in PubMed.

**Search Used for PubMed:**  (((Medical OR health OR healthcare OR health care) AND (Ethic* OR Bioethic* OR Moral* OR Immoral OR Unethic*)) AND (Interpret* OR Translat*)) AND (Language OR Deaf) AND (english[Filter]))

**Search Used for MLA International Bibliography:** (((Medical OR health OR healthcare OR health care) AND (Ethic* OR Bioethic* OR Moral* OR Immoral OR Unethic*)) AND (Interpret* OR Translat*)) AND (Language OR Deaf)))

# **S4 Appendix: Gray Literature Search Strategy**

The gray literature search strategy was developed in consultation with Colleen Foy, MLIS from Z. Smith Reynolds Library. Websites of interpreter societies, government entities with health oversight, and healthcare blogs were identified in consultation with trained interpreters, a reference librarian, and independent research. Websites that did not have credible authors and information were excluded, as well as ones that were inaccessible.

Complex search strings were used on [Google.com](http://google.com) to search through interpreting websites for relevant documents to be included for review on March 6, 2024. Specifically, the following search term was used for each website: site:xxxxx.org "medical" OR "medical interpreting" OR "medical translating" OR "deaf communication" OR "healthcare" OR “language barrier” OR “healthcare translation” OR “medical translation” AND “ethics”. Results were reviewed by the screeners who used guidelines similar to the Title and Abstract screening. Items that met these initial criteria were added to the Zotero group library, tagged as gray literature, and then imported into Covidence (also being tagged there as gray literature) as sources for further full-text review and then, if included, for data extraction.

Eighteen websites were reviewed, from which 16 were included in the study (see Table S3). Websites that were excluded were those that were either inaccessible at the time of search (*n*=1) or yielded no relevant results to medical interpreters or relevant ethical issues (*n*=1).

**S4 Table. Gray Literature Websites Assessed.** Eighteen websites were assessed as part of the gray literature review with 16 of them included in the final assessment.

| **#** | **Organization** | **Website** | **Included** |
| --- | --- | --- | --- |
| 1 | American Foundation for Translation and Interpretation | [afti.org](http://afti.org) | No |
| 2 | American Translators Association | [atanet.org](http://atanet.org) | Yes |
| 3 | World Association of Sign Language Interpreters | [wasli.org](http://wasli.org) | Yes |
| 4 | American Translation and Interpreting Studies Association | [atisa.org](http://atisa.org) | Yes |
| 5 | International Association for Translation and Intercultural Studies | [iatis.org](http://iatis.org) | Yes |
| 6 | International Federation of Translators | [fit-ift.org](http://fit-ift.org) | Yes |
| 7 | Translators Without Borders | [translatorswithoutborders.org](http://translatorswithoutborders.org) | Yes |
| 8 | New York Circle of Translators | [nyctranslators.org](http://nyctranslators.org) | Yes |
| 9 | National Association of Judiciary Interpreters and Translators | [najit.org](http://najit.org) | Yes |
| 10 | American Literary Translators Association | [literarytranslators.org](http://literarytranslators.org) | No |
| 11 | Registry of Interpreters for the Deaf, Inc. | [rid.org](http://rid.org) | Yes |
| 12 | National Board of Certification for Medical Interpreters | [certifiedmedicalinterpreters.org](http://certifiedmedicalinterpreters.org) | Yes |
| 13 | International Medical Interpreters Association | [imiaweb.org](http://imiaweb.org) | Yes |
| 14 | US Department of Health and Human Services, Office of Civil Rights | [hhs.gov](http://hhs.gov) | Yes |
| 15 | US Department of Health and Human Services, Office of Minority Health | [minorityhealth.hhs.gov](http://minorityhealth.hhs.gov) | Yes |
| 16 | National Academies of Science, Engineering, and Medicine | [nationalacadamies.org](http://nationalacadamies.org) | Yes |
| 17 | National Institutes of Health - Office of Diversity, Equity, and Inclusion* | [www.edi.nih.gov](http://www.edi.nih.gov) | Yes |
| 18 | Office of Global Health* | [www.hrsa.gov/office-global-health](http://www.hrsa.gov/office-global-health) | Yes |

*Note: *These offices no longer exist on the government websites due to government restructuring in spring 2025.*

# **S5 Appendix. Literature Analyzed in the Study**.

**S5 Table. Literature Analyzed.** 112 studies were assessed as part of the systematic mapping of literature.

| **##** | **Citation** | **Article Type** | **Ethical Issues** | **Roles** |
| --- | --- | --- | --- | --- |
| **1** | Testai M. Voices from The Clinic: Interpreters, Patients, and Power. Narrative Inquiry in Bioethics. 2024;14(3):195–200. doi: 10.1353/nib.2024.a947861. | Case Series | Accuracy; Neutrality; Interprofessionalism; Emotional or Moral Distress | Linguistic Conduit; Cultural Broker; Patient Advocate |
| **2** | Goler E, Mack JW. Ethical Tensions in the Role of the Medical Interpreter. Narrative Inquiry in Bioethics. 2024 Jan 1;14(3):189–93. doi: 0.1353/nib.2024.a947860. | Case Series | Accuracy; Neutrality; Interprofessionalism; Emotional or Moral Distress | Linguistic Conduit; Patient Advocate |
| **3** | Epstein B. Learning the Language of Medical Interpreters. Narrative Inquiry in Bioethics. 2024;14(3):175–81. doi: 10.1353/nib.2024.a947858. | Case Series | Accuracy; Neutrality; Interprofessionalism; Emotional or Moral Distress | Linguistic Conduit; Patient Advocate |
| **4** | Meyer C. 1-800-Quit-Now. Narrative Inquiry in Bioethics. 2024;14(3):E12–3.  doi: 10.1353/nib.2024.a947870. | Case Series | Accuracy; Neutrality | Linguistic Conduit; Cultural Broker; Patient Advocate |
| **5** | Coronado P. Being an Interpreter—Beyond Linguistics. Narrative Inquiry in Bioethics. 2024;14(3):E10–2. doi: 10.1353/nib.2024.a947869. | Case Series | Accuracy; Neutrality | Linguistic Conduit; Cultural Broker |
| **6** | Milkowska-Shibata M. Witnessing Trauma: Emotional Challenges in Medical Interpretation. Narrative Inquiry in Bioethics. 2024;14(3):E8–10. doi: 10.1353/nib.2024.a947868. | Case Series | Neutrality; Professionalism; Emotional or Moral Distress | Linguistic Conduit; Patient Advocate |
| **7** | Moreno R.C. To Engage or Not to Engage: An Interpreter and a Mother’s Need for Connection in the Cardiothoracic Unit. Narrative Inquiry in Bioethics. 2024;14(3):E7–8.  doi: 10.1353/nib.2024.a947867. | Case Series | Neutrality; Confidentiality; Interprofessionalism | Linguistic Conduit; Cultural Broker |
| **8** | Rutherford-Rojas M. And When May I Cry? Juggling Emotions in Healthcare Interpreting. Narrative Inquiry in Bioethics. 2024;14(3):E6–7. doi: 10.1353/nib.2024.a947866. | Case Series | Neutrality; Emotional or Moral Distress | Cultural Broker |
| **9** | Cooper K, Russell M, Chaiken D, Mazzaroppi M.W., Roman G. How Policies and Practices in Medical Settings Impact Communication Access with Deaf Patients and Caregivers. Narrative Inquiry in Bioethics. 2024 Jan 1;14(3):E3–6. doi: 10.1353/nib.2024.a947865. | Case Series | Neutrality; Time Constraints | Cultural Broker |
| **10** | Pollack-Johnson L. The Ramp and the Stop Sign. Narrative Inquiry in Bioethics. 2024;14(3):E1–3.doi: 10.1353/nib.2024.a947864. | Case Series | Accuracy | Linguistic Conduit |
| **11** | DeSouza L. The Voice of Patients: The Exclusive Work of a Human Who Can Advocate. Narrative Inquiry in Bioethics. 2024;14(3):170–1. doi: 10.1353/nib.2024.a947856. | Case Series | Neutrality; Emotional or Moral Distress | Patient Advocate |
| **12** | Ricci N. Don’t Mute the Messenger. Narrative Inquiry in Bioethics. 2024;14(3):171–4. doi: 10.1353/nib.2024.a947857. | Case Series | Neutrality; Confidentiality; Interprofessionalism; Emotional or Moral Distress | Cultural Broker; Patient Advocate |
| **13** | Crane L. Voices in the Shadows: The Hidden Complexities of Being a Medical Interpreter. Narrative Inquiry in Bioethics. 2024;14(3):167–70. doi: 10.1353/nib.2024.a947855. | Case Series | Neutrality; Interprofessionalism; Emotional or Moral Distress | Cultural Broker; Patient Advocate |
| **14** | Almazan L. Translating Care for the Voiceless Patient. Narrative Inquiry in Bioethics. 2024;14(3):152–4. doi: 10.1353/nib.2024.a947849. | Case Series | Neutrality; Interprofessionalism | Linguistic Conduit; Patient Advocate |
| **15** | Ma Y. “Call me Dr. XXX!” Narrative Inquiry in Bioethics. 2024;14(3):151–2. doi: 10.1353/nib.2024.a947848. | Case Series | Interprofessionalism; Professionalism | Cultural Broker |
| **16** | Hostovsky P. Deaf Interpreter. Narrative Inquiry in Bioethics. 2024;14(3):148–50. doi: 10.1353/nib.2024.a947847. | Case Series | Accuracy; Neutrality | Cultural Broker |
| **17** | O’Leary G. A Day in the Life of a Spanish Interpreter. Narrative Inquiry in Bioethics. 2024;14(3):146–8. doi: 10.1353/nib.2024.a947846. | Case Series | Accuracy; Time Constraints | Linguistic Conduit |
| **18** | Goldhirsch J, Scott N, Ma Y, Abrahm J. Addressing Vicarious Trauma and Emotional Stress Among Medical Interpreters Through Care, Partnership and Respect. New Voices in Translation Studies. 2022;27(1):119–41. doi: 10.14456/nvts.2022.24.16. | Case Report | Accuracy; Neutrality; Interprofessionalism; Emotional or Moral Distress | Linguistic Conduit; Cultural Broker |
| **19** | Waters A.R., Zamora E.R., Fluchel M, Warner E.L., Rosen S, Gwilliam V, et al. A Qualitative Inquiry of Communication Based Barriers to the Diagnosis of Pediatric Cancer: Perceptions of Primarily Spanish-speaking Caregivers. Patient Education and Counseling. 2021 Sep. doi: 10.1016/j.pec.2021.09.028. | Qualitative Research | Neutrality; Interprofessionalism | Linguistic Conduit; Cultural Broker; Patient Advocate |
| **20** | White R.G., Fay R, Chiumento A, Giurgi-Oncu C, Phipps A. Communication about distress and well-being: Epistemic and ethical considerations. Transcultural Psychiatry. 2022 Mar 18;136346152210827. doi: 10.1177/13634615221082795. | Text and Opinion | Accuracy; Neutrality; Professionalism; Emotional or Moral Distress | Linguistic Conduit; Patient Advocate |
| **21** | Wiking E, Saleh-Stattin N, Johansson S.E ., Sundquist J. A description of some aspects of the triangular meeting between immigrant patients, their interpreters and GPs in primary health care in Stockholm, Sweden. Family Practice. 2009 Aug 27;26(5):377–83. doi: 10.1093/fampra/cmp052. | Qualitative Research | Accuracy; Interprofessionalism | Linguistic Conduit; Cultural Broker; Patient Advocate |
| **22** | Wendler D, Cornelio M. Overcoming language barriers in medical care. Pediatric Blood & Cancer. 2006 Nov;47(6):747–7. doi: 10.1002/pbc.20862. | Case Report | Accuracy | Linguistic Conduit |
| **23** | White J, Plompen T, Osadnik C, Tao L, Micallef E, Haines T. The experience of interpreter access and language discordant clinical encounters in Australian health care: a mixed methods exploration. International Journal for Equity in Health. 2018 Sep 24;17(1). doi: 10.1186/s12939-018-0865-2. | Qualitative Research | Accuracy; Interprofessionalism; Time Constraints | Linguistic Conduit |
| **24** | Wu M.S., Rawal S. “It’s the difference between life and death”: The views of professional medical interpreters on their role in the delivery of safe care to patients with limited English proficiency. Brown S, editor. PLOS ONE. 2017 Oct 5;12(10):e0185659. doi: 10.1371/journal.pone.0185659.41. | Qualitative Research | Accuracy; Neutrality; Professionalism; Emotional or Moral Distress | Linguistic Conduit; Patient Advocate |
| **25** | Würth K.M., Reiter-Theil S, Langewitz W, Schuster S. “Getting by” in a Swiss Tertiary Hospital: the Inconspicuous Complexity of Decision-making Around Patients’ Limited Language Proficiency. Journal of General Internal Medicine. 2018 Aug 24;33(11):1885–91. doi: 10.1007/s11606-018-4618-0. | Qualitative Research | Accuracy; Interprofessionalism | Linguistic Conduit |
| **26** | Schenker Y, Smith A.K., Arnold R.M., Fernandez A. “Her Husband Doesn’t Speak Much English”: Conducting a Family Meeting with an Interpreter. Journal of Palliative Medicine. 2012 Apr;15(4):494–8. doi: 10.1089/jpm.2011.0169. | Case Report | Accuracy; Neutrality; Confidentiality; Interprofessionalism; Professionalism; Emotional or Moral Distress | Linguistic Conduit |
| **27** | Schenker Y, Fernandez A, Kerr K, O’Riordan D, Pantilat S.Z. Interpretation for Discussions about End-of-Life Issues: Results from a National Survey of Health Care Interpreters. Journal of Palliative Medicine. 2012 Sep;15(9):1019–26. doi: 10.1089/jpm.2012.0032. | Qualitative Research | Interprofessionalism; Professionalism; Emotional or Moral Distress | Linguistic Conduit |
| **28** | Schenker Y, Wang F, Selig S.J., Ng R, Fernandez A. The Impact of Language Barriers on Documentation of Informed Consent at a Hospital with On-Site Interpreter Services. Journal of General Internal Medicine. 2017 Oct 24;22(S2):294–9. doi: 10.1007/s11606-007-0359-1. | Non-randomised Experimental Study | Interprofessionalism | Linguistic Conduit |
| **29** | Schreiber J.R., Odell M.S. The use of interpreters in medical settings and forensic medical examinations in Australia: The relationship between medicine and linguistics. Journal of Forensic and Legal Medicine. 2014 Jul;25:85–90. doi: 10.1016/j.jflm.2014.04.019. | Case Report | Accuracy; Neutrality; Confidentiality; Interprofessionalism | Linguistic Conduit; Patient Advocate |
| **30** | Seale C, Rivas C, Al-Sarraj H, Webb S, Kelly M. Moral mediation in interpreted health care consultations. Social Science & Medicine. 2013 Dec;98:141–8. doi: 10.1016/j.socscimed.2013.09.014. | Qualitative Research | Neutrality; Interprofessionalism; Professionalism | Linguistic Conduit; Patient Advocate |
| **31** | Sellevold V, Hamre L, Bondas T. A meta-ethnography of Language Challenges in Midwifery Care. European Journal of Midwifery. 2022 Jul 5;6(41):1–13. doi: 10.18332/ejm/147994. | Systematic Review | Accuracy; Confidentiality; Interprofessionalism | Linguistic Conduit; Cultural Broker; Patient Advocate |
| **32** | Torkpoor R, Fioretos I, Essén B, Londos E. “I Know Hyena. Do you Know Hyena?” Challenges in Interpreter-Mediated Dementia Assessment, Focusing on the Role of the Interpreter. Journal of Cross-Cultural Gerontology. 2022 Mar;37(1):45–67. doi: 10.1007/s10823-021-09439-7. | Qualitative Research | Accuracy; Interprofessionalism | Linguistic Conduit |
| **33** | Uhlin K, Persson E, Stålnacke B.M., Löfgren M. Healthcare professionals’ experiences and perspectives of team-based interdisciplinary pain rehabilitation with immigrants requiring an interpreter. A qualitative study. Disability and Rehabilitation. 2022 Aug 23;45(15):1–12.  doi: 10.1080/09638288.2022.2111608. | Qualitative Research | Neutrality; Interprofessionalism; Time Constraints; Professionalism; Emotional or Moral Distress | Linguistic Conduit; Cultural Broker; Patient Advocate |
| **34** | Van Cleave A.C., Roosen-Runge M.U., Miller A.B., Milner L.C., Karkazis K.A., Magnus D.C.. Quality of Communication in Interpreted Versus Noninterpreted PICU Family Meetings. Critical Care Medicine. 2014 Jun;42(6):1507–17.  42(6), 1507–1517. doi: 10.1097/CCM.0000000000000177. | Qualitative Research | Interprofessionalism | Linguistic Conduit |
| **35** | Vernon M, Miller K. Interpreting in Mental Health Settings: Issues and Concerns. American Annals of the Deaf. 2001;146(5):429–34. doi: 10.1353/aad.2012.0200. | Case Report | Accuracy; Neutrality; Confidentiality; Professionalism | Linguistic Conduit |
| **36** | Pechak C, Summers C, Velasco J. Improved Knowledge Following an Interprofessional Interpreter-Use Training. Journal of Allied Health. 2018 Fall;47(3): 159–166. | Non-random Experimental Study | Interprofessionalism; Time Constraints | Linguistic Conduit |
| **37** | Prentice J, Nelson A, Baillie J, Osborn H, Noble S. “Don’t blame the middle man”: an exploratory qualitative study to explore the experiences of translators breaking bad news. Journal of the Royal Society of Medicine. 2014 Mar 18;107(7):271–6. doi: 10.1177/0141076814527275.35. | Qualitative Research | Neutrality; Interprofessionalism; Professionalism; Emotional or Moral Distress | Linguistic Conduit; Patient Advocate |
| **38** | Quesada P.R., Solis R.N., Levi J.R. Student Medical Interpreters: A Double-Edged Sword. Otolaryngology–Head and Neck Surgery. 2020 Mar 10;162(4):476–8. doi: 10.1177/0194599820909083. | Qualitative Research | Accuracy; Neutrality | Linguistic Conduit; Cultural Broker |
| **39** | Ragavan MI, Cowden JD. The Complexities of Assessing Language and Interpreter Preferences in Pediatrics. Health Equity. 2018 Jun;2(1):70–3. doi: 10.1089/heq.2017.0057. | Case Report | Accuracy; Neutrality; Confidentiality; Time Constraints | Linguistic Conduit; Cultural Broker |
| **40** | Ralph A.P., Lowell A, Murphy J, Dias T, Butler D, Spain B, et al. Low uptake of Aboriginal interpreters in healthcare: exploration of current use in Australia’s Northern Territory. BMC Health Services Research. 2017 Nov 15;17(1). doi: 10.1186/s12913-017-2689-y. | Non-randomised Experimental Study | Accuracy; Neutrality; Time Constraints | Linguistic Conduit; Cultural Broker |
| **41** | Mecham J.C., Marisa Martinez Salazar, Perez R, Castaneda U, Beatriz Garcia Stamps, Chavez A, et al. Exploring the Factors that Influence Ethical Spanish Use among Medical Students and Solutions for Improvement. Teaching and Learning in Medicine. 2021 Jul 27;34(5):522–9.  doi: 10.1080/10401334.2021.1949996. | Qualitative Research | Accuracy; Time Constraints | Linguistic Conduit |
| **42** | Meddings F, Haith-Cooper M. Culture and Communication in Ethically Appropriate Care. Nursing Ethics. 2008 Jan;15(1):52–61. doi: 10.1177/0969733007083934. | Case Report | Neutrality; Professionalism | Cultural Broker |
| **43** | Odhiambo R, Mars M. Patients’ understanding of telemedicine terms required for informed consent when translated into Kiswahili. BMC Public Health. 2018 May 3;18(1). doi: 10.1186/s12889-018-5499-1. | Non-randomised Experimental Study | Accuracy; Professionalism | Linguistic Conduit; Cultural Broker |
| **44** | Olson A.M., Swabey L. Communication Access for Deaf People in Healthcare Settings. Journal for Healthcare Quality. 2017;39(4):191–9. doi: 10.1097/JHQ.0000000000000038. | Non-randomised Experimental Study | Accuracy; Neutrality; Interprofessionalism; Emotional or Moral Distress | Linguistic Conduit; Cultural Broker |
| **45** | Lundin C, Hadziabdic E, Hjelm K. Language interpretation conditions and boundaries in multilingual and multicultural emergency healthcare. BMC International Health and Human Rights. 2018 Jun 5;18(1). doi: 10.1186/s12914-018-0157-3.30. | Qualitative Research | Interprofessionalism; Time Constraints; Professionalism | Linguistic Conduit |
| **46** | Mangrio E, Sjögren Forss K. Refugees’ experiences of healthcare in the host country: a scoping review. BMC Health Services Research. 2017 Dec;17(1). doi: 10.1186/s12913-017-2731-0. | Systematic Review | Confidentiality; Interprofessionalism; Time Constraints | Linguistic Conduit; Cultural Broker |
| **47** | Matiasek J, Wynia M.K. Reconceptualizing the Informed Consent Process at Eight Innovative Hospitals. The Joint Commission Journal on Quality and Patient Safety. 2008 Mar;34(3):127–37. doi: 10.1016/s1553-7250(08)34015-x. | Non-randomised Experimental Study | Interprofessionalism; Time Constraints; Professionalism | Linguistic Conduit |
| **48** | Maul L, Regenstein M, Andres E, Wright R, Wynia M.K. Using A Risk Assessment Approach to Determine Which Factors Influence Whether Partially Bilingual Physicians Rely on Their Non-English Language Skills or Call an Interpreter. The Joint Commission Journal on Quality and Patient Safety. 2012 Jul;38(7):328–36. doi: 10.1016/s1553-7250(12)38043-4. | Qualitative Research | Accuracy; Interprofessionalism; Time Constraints | Linguistic Conduit |
| **49** | McGrath P, Holewa H, Ogilvie K, Rayner R, Patton M.A. Insights on Aboriginal peoples’ views of cancer in Australia. Contemporary Nurse. 2006 Sep;22(2):240–54. doi: 10.5172/conu.2006.22.2.240. | Non-randomised Experimental Study | Accuracy | Linguistic Conduit |
| **50** | Joseph G, Lindberg N.M., Guerra C, Hernandez C, Karliner L.S., Gilmore M.J., et al. Medical interpreter‐mediated genetic counseling for Spanish preferring adults at risk for a hereditary cancer syndrome. Journal of Genetic Counseling. 2023 Mar 20;32(4):870–86. doi: 10.1002/jgc4.1695. | Non-randomised Experimental Study | Accuracy; Interprofessionalism; Professionalism | Linguistic Conduit; Cultural Broker |
| **51** | Kalengayi F.K.N., Hurtig A.K., Nordstrand A, Ahlm C, Ahlberg B.M. “It is a dilemma”: perspectives of nurse practitioners on health screening of newly arrived migrants. Global Health Action. 2015 Jul 22;8(1):27903. doi: 10.3402/gha.v8.27903. | Qualitative Research | Accuracy; Time Constraints | Linguistic Conduit |
| **52** | Kaufert J.M., Putsch R.W. Communication through Interpreters in Healthcare: Ethical Dilemmas Arising from Differences in Class, Culture, Language, and Power. The Journal of Clinical Ethics. 1997 Mar 1;8(1):71–87.24. | Case Report | Accuracy; Neutrality; Confidentiality; Professionalism; Emotional or Moral Distress | Linguistic Conduit; Cultural Broker; Patient Advocate |
| **53** | Kastrup M. Ethical aspects in providing care to marginalized populations. International Review of Psychiatry. 2010 Jun;22(3):252–7. doi: 10.3109/09540261.2010.484015. | Case Report | Neutrality; Confidentiality | Cultural Broker; Patient Advocate |
| **54** | Kletečka-Pulker M, Parrag S, Doppler K, Völkl-Kernstock S, Wagner M, Wenzel T. Enhancing patient safety through the quality assured use of a low-tech video interpreting system to overcome language barriers in healthcare settings. Wiener klinische Wochenschrift. 2021 Feb 2;133(11-12):610–9. doi: 10.1007/s00508-020-01806-7. | Non-randomised Experimental Study | Interprofessionalism | Linguistic Conduit |
| **55** | Le Neveu M, Berger Z, Gross M. Lost in Translation: the Role of Interpreters on Labor and Delivery. Health Equity. 2020 Sep 1;4(1):406–9. doi: 10.1089/heq.2020.0016. | Qualitative Research | Accuracy; Time Constraints | Patient Advocate |
| **56** | Lehna C. Interpreter services in pediatric nursing. Pediatric Nursing. 2005 Jul-Aug;31(4):292–296. | Case Report | Accuracy; Neutrality; Confidentiality; Professionalism | Linguistic Conduit; Cultural Broker; Patient Advocate |
| **57** | Leonard R, Paton J, Hinton P, Greenaway S, Thomson J. The end-of-life needs of Aboriginal and immigrant communities: a challenge to conventional medical models. Frontiers in Public Health. 2023 Jul 19;11(1):1161267. doi: 10.3389/fpubh.2023.1161267. | Non-randomised Experimental Study | Accuracy; Neutrality; Interprofessionalism; Emotional or Moral Distress | Linguistic Conduit; Cultural Broker |
| **58** | Lim C, Zhou J.X., Woong NL, Chiam M, Krishna L.K.R. Addressing the Needs of Migrant Workers in ICUs in Singapore. Journal of Medical Education and Curricular Development. 2020 Jan;7. doi: 10.1177/2382120520977190. | Qualitative Research | Accuracy; Neutrality; Confidentiality | Linguistic Conduit; Cultural Broker |
| **59** | Gutierrez A.M., Statham E.E., Robinson J.O., Slashinski M.J., Scollon S, Bergstrom K.L., et al. Agents of empathy: How medical interpreters bridge sociocultural gaps in genomic sequencing disclosures with Spanish-speaking families. Patient Education and Counseling. 2019 May;102(5):895–901. doi: 10.1016/j.pec.2018.12.012. | Non-randomised Experimental Study | Accuracy; Neutrality | Linguistic Conduit; Cultural Broker; Patient Advocate |
| **60** | Hancox J. A., McKiernan C. F., Martin A. L., Tomas J., MacArtney J. I. The emotional effects on professional interpreters of interpreting palliative care conversations for adult patients: A rapid review. Palliative Medicine. 2023 Apr 24;*37*(7): 931–946. doi: 10.1177/02692163231169318. | Systematic Review | Neutrality; Professionalism; Emotional or Moral Distress | Linguistic Conduit; Patient Advocate |
| **61** | Hayes B, Fabri A.M., Coperchini M, Parkar R, Austin-Crowe Z. Health and death literacy and cultural diversity: insights from hospital-employed interpreters. BMJ Supportive & Palliative Care. 2017 Jul 4;10(1):e8–8. doi: 10.1136/bmjspcare-2016-001225. | Qualitative Research | Accuracy; Neutrality; Emotional or Moral Distress | Linguistic Conduit; Cultural Broker |
| **62** | Hordyk S.R., Macdonald M.E., Brassard P. Inuit interpreters engaged in end-of-life care in Nunavik, Northern Quebec. International Journal of Circumpolar Health. 2017 Jan;76(1).  doi: 10.1080/22423982.2017.1291868. | Non-randomised Experimental Study | Accuracy; Neutrality; Professionalism; Emotional or Moral Distress | Linguistic Conduit; Cultural Broker; Patient Advocate |
| **63** | Hsieh E. Interpreters as co-diagnosticians: Overlapping roles and services between providers and interpreters. Social Science & Medicine. 2007 Feb;64(4):924–37.  doi: 10.1016/j.socscimed.2006.10.015. | Qualitative Research | Accuracy; Neutrality; Emotional or Moral Distress | Cultural Broker; Patient Advocate |
| **64** | Hsieh E. Not Just “Getting by”: Factors Influencing Providers’ Choice of Interpreters. Journal of General Internal Medicine. 2014 Oct 23;30(1):75–82. doi: 10.1007/s11606-014-3066-8. | Qualitative Research | Accuracy; Time Constraints | Linguistic Conduit; Patient Advocate |
| **65** | Hsieh E, Kramer E.M. Medical interpreters as tools: Dangers and challenges in the utilitarian approach to interpreters’ roles and functions. Patient Education and Counseling. 2012 Oct;89(1):158–62. doi: 10.1016/j.pec.2012.07.001. | Qualitative Research | Interprofessionalism; Time Constraints; Emotional or Moral Distress | Linguistic Conduit; Patient Advocate |
| **66** | Huang A.J.R., Siyambalapitiya S, Cornwell P. Speech pathologists and professional interpreters managing culturally and linguistically diverse adults with communication disorders: a systematic review. International Journal of Language & Communication Disorders. 2019 May 22;54(5):689–704. doi: 10.1111/1460-6984.12475. | Systematic Review | Interprofessionalism; Time Constraints | Linguistic Conduit; Cultural Broker |
| **67** | Dysart-Gale D. Communication Models, Professionalization, and the Work of Medical Interpreters. Health Communication. 2005 Jan;17(1):91–103.  doi: 10.1207/s15327027hc1701_6. | Case Report | Accuracy; Neutrality; Confidentiality; Emotional or Moral Distress | Linguistic Conduit; Cultural Broker; Patient Advocate |
| **68** | Emmerich N, Phillips C. Should professional interpreters be able to conscientiously object in healthcare settings? Journal of Medical Ethics. 2019 Dec 3. doi: 10.1136/medethics-2019-105767.13. | Systematic Review | Accuracy; Neutrality; Professionalism; Emotional or Moral Distress | Linguistic Conduit; Cultural Broker |
| **69** | Espinoza Suarez N.R., Urtecho M, Nyquist C.A., Jaramillo C, Yeow M.E., Thorsteinsdottir B, et al. Consequences of suboptimal communication for patients with limited English proficiency in the intensive care unit and suggestions for a way forward: A qualitative study of healthcare team perceptions. Journal of Critical Care. 2021 Feb;61:247–51. doi: 10.1016/j.jcrc.2020.10.012. | Qualitative Research | Neutrality; Interprofessionalism; Emotional or Moral Distress | Linguistic Conduit; Cultural Broker |
| **70** | Goldhirsch J, Halpenny B, Scott N, Ma Y, Rodriguez MS, Abrahm JL. What’s Lost in Translation: A Dialogue-Based Intervention That Improves Interpreter Confidence in Palliative Care Conversations. Journal of Pain and Symptom Management. 2021 Sep;62(3):609–14.  doi: 10.1016/j.jpainsymman.2021.02.027. | Qualitative Research | Accuracy; Interprofessionalism; Emotional or Moral Distress | Cultural Broker; Patient Advocate |
| **71** | Green A.R., Nze C. Language-Based Inequity in Health Care: Who Is the “Poor Historian”?. AMA journal of ethics. 2017;19(3):263–71. doi: 10.1001/journalofethics.2017.19.3.medu1-1703. | Case Report | Accuracy; Interprofessionalism; Time Constraints; Professionalism | Linguistic Conduit; Patient Advocate |
| **72** | Bamford J, Abraham S, Alachkar M, Akinola A. Falling on deaf ears: interpreters as cultural brokers in mental healthcare. BJPsych bulletin. 2024 Jan 5;48(2):73–7. doi: 10.1192%2Fbjb.2023.90. | Qualitative Research | Neutrality; Confidentiality | Linguistic Conduit; Cultural Broker |
| **73** | Barwise A, Sharp R, Hirsch J. Ethical Tensions Resulting from Interpreter Involvement in the Consent Process. Ethics & Human Research. 2019 Jul;41(4):31–5. doi: 10.1002/eahr.500025. | Case Report | Interprofessionalism; Professionalism | Cultural Broker; Patient Advocate |
| **74** | Bibler T, Peña A, Bruce CR. Consultations across Languages. Hastings Center Report. 2015 May;45(3):13–4. doi: 10.1002/hast.444. | Case Report | Neutrality; Interprofessionalism; Time Constraints; Emotional or Moral Distress | Cultural Broker; Patient Advocate |
| **75** | Birkelund L, Dieperink K.B., Sodemann M, Lindell J.F., Steffensen K.D., Nielsen D.S. Communicating without a shared language: A qualitative study of language barriers in language-discordant cancer communication. Journal of Health Communication. 2024 Jan 31;29(3):1–13. doi: 10.1080/10810730.2024.2309357. | Qualitative Research | Accuracy; Interprofessionalism; Time Constraints | Linguistic Conduit |
| **76** | Boness C.L. Treatment of Deaf Clients: Ethical Considerations for Professionals in Psychology. Ethics & Behavior. 2016;26(7):562–85. doi: 10.1080/10508422.2015.1084929. | Text and Opinion | Accuracy; Neutrality; Interprofessionalism; Professionalism | Linguistic Conduit; Cultural Broker |
| **77** | Brijnath B, Gonzalez E, Hlavac J, Enticott J, Woodward‐Kron R, LoGiudice D, et al. The impact of training on communication quality during interpreter‐mediated cognitive assessments: Study protocol for a randomized controlled trial. Alzheimer’s & Dementia: Translational Research & Clinical Interventions. 2022 Jan;8(1). doi: 10.1002/trc2.12349. | Randomized Controlled Trial | Accuracy; Professionalism | Linguistic Conduit |
| **78** | Clifford A. Is Fidelity Ethical? TTR : traduction, terminologie, rédaction. 2006 Jul 20;17(2):89–114. doi: 10.7202/013273ar. | Text and Opinion | Accuracy; Confidentiality | Linguistic Conduit |
| **79** | CHIA Standards. Chiaonline.org. 2016. Available from: https://chiaonline.org/CHIA-Standards | Other | Accuracy; Neutrality; Confidentiality; Interprofessionalism; Time Constraints; Professionalism | Linguistic Conduit; Cultural Broker; Patient Advocate |
| **80** | Cortabarria B. The role of translators and interpreters in hybrid English–Spanish contexts in the USA. Language and Intercultural Communication. 2015 Mar 13;15(3):407–23. doi:  10.1080/14708477.2015.1015345. | Qualitative Research | Accuracy; Professionalism; Emotional or Moral distress | Cultural Broker; Patient Advocate |
| **81** | Ineke H.M. Crezee, Jülich S. Chapter 9. Exploring role expectations of healthcare interpreters in New Zealand. Benjamin's Translation Library. 2020 May 12;211–41. doi: 10.1075/btl.151.09cre. | Qualitative Research | Accuracy; Neutrality; Confidentiality; Emotional or Moral distress | Linguistic Conduit; Cultural Broker; Patient Advocate |
| **82** | Crezee I, Marianacci A. “How did he say that?” interpreting students’ written reflections on interprofessional education scenarios with speech language therapists. The Interpreter and Translator Trainer. 2021 Mar 26;1–20. doi: 10.1080/1750399x.2021.1904170. | Qualitative Research | Accuracy; Neutrality; Interprofessionalism | Linguistic Conduit; Cultural Broker |
| **83** | Howes L.M. Ethical dilemmas in community interpreting: interpreters’ experiences and guidance from the code of ethics. The Interpreter and Translator Trainer. 2022 Oct 29;17(2):1–18. doi: 10.1080/1750399x.2022.2141003. | Qualitative Research | Accuracy; Neutrality; Confidentiality; Interprofessionalism; Professionalism; Emotional or Moral distress | Linguistic Conduit; Cultural Broker; Patient Advocate |
| **84** | Ortiz P.M. Multimodal Coordination of Interaction by Interpreters in Bilingual Healthcare Encounters. 2017. | Non-randomised Experimental Study | Accuracy; Neutrality; Confidentiality; Professionalism; Emotional or Moral Distress | Linguistic Conduit; Cultural Broker; Patient Advocate |
| **85** | Swabey L. Interpreting in Healthcare Settings: Gallaudet University Press eBooks. 2018 Sep 28;73–92. | Other | Neutrality; Confidentiality; Interprofessionalism | Linguistic Conduit |
| **86** | Showstack R.E. Making Sense of the Interpreter Role in a Healthcare Service-learning Program. Applied Linguistics. 2020 Feb 22;42(1):93–112. doi: 10.1093/applin/amz058. | Qualitative Research | Accuracy; Neutrality; Professionalism | Linguistic Conduit; Cultural Broker; Patient Advocate |
| **87** | Singy P, Guex P. The interpreter’s role with immigrant patients: Contrasted points of view. Communication Medicine. 2005 Jan;2(1):45–51. doi: 10.1515/come.2005.2.1.45. | Qualitative Research | Accuracy; Neutrality; Interprofessionalism | Linguistic Conduit; Cultural Broker; Patient Advocate |
| **88** | Şener O, Kincal Ş. Türkiye’de Hastane Çevirmenliğinde Rol ve Etik. Çeviribilim ve Uygulamaları Dergisi. 2019 Dec 31;(27):201–19. doi: 10.37599/ceviri.563085. | Case Report | Accuracy; Interprofessionalism; Professionalism | Linguistic Conduit; Patient Advocate |
| **89** | Burkle C.M., Anderson K.A., Xiong Y, Guerra A.E., Tschida-Reuter D.A. Assessment of the efficiency of language interpreter services in a busy surgical and procedural practice. BMC Health Services Research. 2017 Jul 4;17(1). doi: 10.1186/s12913-017-2425-7. | Non-randomised Experimental Study | Time Constraints | Linguistic Conduit |
| **90** | Carlsson T, Balbas B, Mattsson E. Written narratives from immigrants following a prenatal diagnosis: qualitative exploratory study. BMC Pregnancy and Childbirth. 2019 May 6;19(1).  doi: 10.1186/s12884-019-2292-9. | Non-randomised Experimental Study | Accuracy; Interprofessionalism; Time Constraints | Linguistic Conduit |
| **91** | Cassim S, Kidd J, Ali M, Abdul Hamid N, Jamil D, Keenan R, et al. “Look, wait, I’ll translate”: refugee women’s experiences with interpreters in healthcare in Aotearoa New Zealand. Australian Journal of Primary Health. 2022;28(4). doi: 10.1071/py21256. | Qualitative Research | Accuracy; Confidentiality; Emotional or Moral distress | Linguistic Conduit; Patient Advocate |
| **92** | Chatham R.E., Mixer S.J. Methods, Ethics, and Cross-Language Considerations in Research With Ethnic Minority Children. Nursing Research. 2021 Sep;70(5):383–90.  doi: 10.1097/NNR.0000000000000537. | Qualitative Research | Neutrality; Professionalism | Cultural Broker; Patient Advocate |
| **93** | Cheng J.K.Y., Guerra C, Pasick R.J., Schillinger D, Luce J, Joseph G. Cancer genetic counseling communication with low-income Chinese immigrants. Journal of Community Genetics. 2017 Dec 1;9(3):263–76. doi: 10.1007/s12687-017-0350-4. | Non-randomised Experimental Study | Accuracy; Interprofessionalism; Professionalism | Linguistic Conduit |
| **94** | Aitken G. Medical Students as Certified Interpreters. AMA Journal of Ethics. 2019 Mar 1;21(3):E232-238. doi: 10.1001/amajethics.2019.232. | Case Report | Accuracy; Neutrality; Interprofessionalism | Linguistic Conduit |
| **95** | Alnaes A.H. Lost in Translation. Cambridge Quarterly of Healthcare Ethics. 2012 Jul 24;21(4):505–16. doi: 10.1017/s0963180112000278. | Case Report | Confidentiality | Patient Advocate |
| **96** | Ault R, Morales A, Ault R, Spitale A, Martinez G.A. Communication pitfalls in interpreted genetic counseling sessions. Journal of Genetic Counseling. 2019 May 21. doi: 10.1002/jgc4.1132. | Non-randomised Experimental Study | Interprofessionalism | Linguistic Conduit |
| **97** | Simon C.M., Zyzanski S.J., Durand E, Jimenez X, Kodish E.D. Interpreter Accuracy and Informed Consent Among Spanish-speaking Families with Cancer. Journal of Health Communication. 2006 Jun;11(5):509–22. doi: 10.1080/10810730600752043. | Non-randomised Experimental Study | Accuracy; Neutrality; Interprofessionalism | Linguistic Conduit; Cultural Broker |
| **98** | Sinow C.S., Corso I, Lorenzo J, Lawrence K.A., Magnus D.C., Van A.C. Alterations in Spanish Language Interpretation During Pediatric Critical Care Family Meetings. Critical Care Medicine. 2017 Aug 2;45(11):1915–21.doi: 10.1097/ccm.0000000000002650. | Non-randomised Experimental Study | Accuracy; Neutrality | Linguistic Conduit; Patient Advocate |
| **99** | Suarez N.R.E., Urtecho M, Jubran S, Yeow M.E., Wilson M.E., Boehmer K.R., et al. The Roles of medical interpreters in intensive care unit communication: A qualitative study. Patient Education and Counseling. 2020 Oct;104(5). doi: 10.1016/j.pec.2020.10.018. | Qualitative Research | Neutrality; Professionalism | Linguistic Conduit; Patient Advocate |
| **100** | Ethics and Standards of Practice. Ncihc.org. 2019. Available from: https://www.ncihc.org/ethics-and-standards-of-practice. | Case Report | Accuracy; Neutrality; Confidentiality; Interprofessionalism; Time Constraints; Professionalism | Linguistic Conduit; Cultural Broker; Patient Advocate |
| **101** | Limited English Proficiency. Lep.gov. 2023. Available from: https://www.lep.gov/sites/lep/files/media/document/2023-11/2023%20Department%20of%20Health%20and%20Humman%20Services%20(HHS)%20Language%20Access%20Plan.pdf | Other | Accuracy; Time Constraints; Professionalism | Linguistic Conduit |
| **102** | Hernandez-Iverson E. IMIA Guide on Medical Interpreter Ethical Conduct. 2022 Apr. Available from: https://www.imiaweb.org/uploads/pages/376_2.pdf | Other | Accuracy; Neutrality; Confidentiality; Interprofessionalism; Professionalism | Linguistic conduit; Cultural Broker; Patient Advocate |
| **103** | Nonaka A.M. Legal and Ethical Imperatives for Using Certified Sign Language Interpreters in Health Care Settings: How to “Do No Harm” When “It’s (All) Greek” (Sign Language) to You. Care Management Journals. 2016 Sep 1;17(3):114–28. doi: 10.1891/1521-0987.17.3.114. | Qualitative Research | Accuracy; Confidentiality; Interprofessionalism | Linguistic Conduit |
| **104** | Yang C, Prokop L, Barwise A. Strategies Used by Healthcare Systems to Communicate with Hospitalized Patients and Families with Limited English Proficiency During the COVID-19 Pandemic: A Narrative Review. Journal of Immigrant and Minority Health. 2023 Feb 23;25.  doi: 10.1007/s10903-023-01453-w. | Systematic Review | Accuracy; Interprofessionalism | Linguistic Conduit |
| **105** | Connecting Cultures Healthcare Interpreters. Code of Ethics: An Introduction. YouTube. 2019. Available from: https://www.youtube.com/watch?v=C1r2FPwDELc. | Other | Accuracy; Neutrality; Confidentiality; Interprofessionalism; Professionalism | Linguistic Conduit; Cultural Broker; Patient Advocate |
| **106** | Sanchez-Herrera H. Are We There Yet? A Narrative of Firsthand Interpreter Experiences in the Medical Field and Insights to Aid Language Access Compliance. Narrative Inquiry in Bioethics. 2024 Jan 1;14(3):154–6. doi: 10.1353/nib.2024.a947850. | Case Series | Neutrality; Confidentiality; Interprofessionalism; Professionalism; Emotional or Moral distress | Linguistic conduit; Patient advocate |
| **107** | Will M.R. One Interpreter’s Journey of Interpreting for Pregnancy Loss. Narrative Inquiry in Bioethics. 2024;14(3):156–9. doi: 10.1353/nib.2024.a947851. | Case Series | Accuracy; Neutrality; Professionalism; Emotional of Moral Distress | Linguistic conduit |
| **108** | Ratway F. Towards Language Justice: A Call to Identify and Overcome Structural Barriers. Narrative Inquiry in Bioethics. 2024;14(3):164–7. doi: 10.1353/nib.2024.a947854. | Case Series | Accuracy; Time constraints; Professionalism | Linguistic conduit |
| **109** | McMillan G. Introduction: Stories From Those Who Interpret For Others in Healthcare. Narrative Inquiry in Bioethics. 2024;14(3):143–6. doi: 10.1353/nib.2024.a947845. | Case Series | Accuracy; Neutrality; Confidentiality; Interprofessionalism; Professionalism; Emotional or Moral Distress | Linguistic conduit; Cultural broker; Patient advocate |
| **110** | Hsieh E. More Than Words: Communicating for the Quality of Care. Narrative Inquiry in Bioethics. 2024;14(3):159–61. doi: 10.1353/nib.2024.a947852. | Case Series | Accuracy; Neutrality; Professionalism | Linguistic conduit; Cultural broker; Patient advocate |
| **111** | Gumer J.M. Giving Voice to the Voiceless—Stories of Medical Interpreters. Narrative Inquiry in Bioethics. 2024;14(3):183–7. doi: 10.1353/nib.2024.a947859. | Case Series | Accuracy; Neutrality; Interprofessionalism; Emotional or Moral Distress | Linguistic conduit; Patient advocate |
| **112** | The Couch - Guidance required: Guidelines for Medical Interpreters. NAJIT. 2019. Available from: https://najit.org/guidelines-for-medical-interpreters/ | Other | Neutrality; Professionalism | Linguistic Conduit |
